# Investigation of 20*S*-hydroxyvitamin D_3_ analogs and their 1α-OH derivatives as potent vitamin D receptor agonists with anti-inflammatory activities

**DOI:** 10.1038/s41598-018-19183-7

**Published:** 2018-01-24

**Authors:** Zongtao Lin, Srinivasa R. Marepally, Emily S. Y. Goh, Chloe Y. S. Cheng, Zorica Janjetovic, Tae-Kang Kim, Duane D. Miller, Arnold E. Postlethwaite, Andrzej T. Slominski, Robert C. Tuckey, Carole Peluso-Iltis, Natacha Rochel, Wei Li

**Affiliations:** 1 Department of Pharmaceutical Sciences, University of Tennessee Health Science Center, Memphis, TN 38163 United States; 2 School of Molecular Sciences, University of Western Australia, Perth, WA 6009 Australia; 3 Department of Dermatology, Comprehensive Cancer Center, Cancer Chemoprevention Program, University of Alabama at Birmingham, Birmingham, AL 35294 United States; 4 Department of Medicine, University of Tennessee Health Science Center, Memphis, TN 38163 United States; 5 Department of Veterans Affairs Medical Center, Memphis, TN 38104 United States; 6 Pathology and Laboratory Medicine Service, VA Medical Center at Birmingham, Birmingham, AL 35294 United States; 7 Department of Integrative Structural Biology, Institute of Genetics and of Molecular and Cellular Biology, Centre National de la Recherche Scientifique, Institut National de la Santé de la Recherche Médicale, Université de Strasbourg, 1 rue Laurent Fries, Illkirch, 67404 France; 8 Present Address: Department of Chemistry, University of Pennsylvania, Philadelphia, PA 19104 United States

## Abstract

20*S*-hydroxyvitamin D_3_ [20*S*(OH)D_3_] is anti-inflammatory and not hypercalcemic, suggesting its potential as a lead compound. In this study, side chain modified 20*S*(OH)D_3_ analogs (**4**, **13**, **23** and **33**) together with their 1α-OH derivatives were synthesized and their metabolism and biological activities tested. **4**, **13** and **23** are good substrates for CYP27B1, enabling enzymatic synthesis of their 1α-OH derivatives **5**, **14** and **24**. However, **33** could not be hydroxylated by CYP27B1 and acts as an inhibitor. All analogs were poorer substrates for CYP24A1 than calcitriol, indicating improved catabolic stability. While the parent analogs showed minimal VDR stimulating activity, their 1α-OH derivatives were potent VDR agonists. **4**, **5**, **14** and **24** significantly upregulated the expression of CYP24A1 at the mRNA level, consistent with their VDR activation abilities and indicating that 1α-hydroxylation is required to produce analogs with strong activity. These analogs have anti-inflammatory activities that are influenced by side chain composition and by 1α-hydroxylation. To understand their molecular interactions with the VDR, 20*S*(OH)D_3_, **4** and **33** were co-crystalized with the VDR ligand binding domain, which revealed subtle differences to the calcitriol-bound receptor. This study demonstrates the potential of the 20*S*(OH)D_3_ scaffold for the development of novel anti-inflammatory agents.

## Introduction

**Figure 1:**
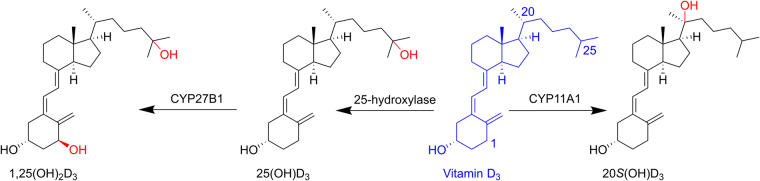
The classical pathway of metabolism of D_3_ to the major form in the circulation, 25(OH)D_3_, and to the active form 1,25(OH)_2_D_3_, and the alternative pathway of metabolism to 20*S*(OH)D_3_.

Vitamin D_3_ (D_3_) can be obtained from either dietary sources through intestinal absorption or endogenous production through dermal synthesis. In the classical pathway of metabolism (Fig. 1), D_3_ which is a prohormone is activated by enzymatic reactions catalyzed by cytochrome P450 (CYP) enzymes in the liver and kidney^1^. The initial step involves 25-hydroxylation in the liver by CYP2R1 to produce 25-hydroxyvitamin D_3_ [25(OH)D_3_] which is the major form of D_3_ in the circulation. The final activation occurs in the kidney where CYP27B1 specifically hydroxylates 25(OH)D_3_ at the 1α position to give 1α,25-dihydroxyvitamin D_3_ [1,25(OH)_2_D_3_], the hormonally (active) form of D_3_^1,2^. Through the vitamin D receptor (VDR), 1,25(OH)_2_D_3_ exerts its effects on mineral homeostasis, as well as displaying anti-inflammatory, anti-proliferative, immunomodulatory, pro-apoptotic and anti-angiogenic activities by modulating the expressions of various VDR target genes, including upregulation of the expression of the gene encoding CYP24A1 which is involved in vitamin D catabolism^1,3,4^.

A novel metabolic pathway (Fig. 1) has been reported by our group for activation and metabolism of D_3_^5,6^ which starts with CYP11A1 acting on D_3_ to produce 20*S*-hydroxyvitamin D_3_ [20*S*(OH)D_3_] as the major product^5,6^, and which operates *in vivo*^7,8^. 20*S*(OH)D_3_ displays many similar activities to that of 1,25(OH)_2_D_3_ including strong anti-proliferative, anti-leukemic, tumorostatic, anti-fibrotic and pro-differentiation activities^9–14^ mediated through either VDR activation^9,15^ or inhibition of RORα and RORγ^16,17^. This metabolite was later shown to cause translocation of the VDR into the nucleus acting as a biased VDR agonist^17,18^, while other studies showed that it also acts as an inverse agonist on RORα and RORγ^16,17^. 20*S*(OH)D_3_ exerts anti-inflammatory activities *in vitro* through inhibition of NFκB and decreasing production of proinflammatory cytokines by keratinocytes^10,15,19,20^, melanoma cells^21^, lymphocytes and macrophages^9,16^, as well as *in vivo* as indicated by suppressive effects on collagen-induced arthritis, at a dose as low as 2 µg/kg in a mouse model^22^. The potency of 20*S*(OH)D_3_ for these anti-inflammatory activities was equal to that of 1,25(OH)_2_D_3_. Also importantly, 20*S*(OH)D_3_ acts as a biased agonist on the VDR, expressing many but not all the properties of 1,25(OH)2D3 and lacks the calcemic (toxic) activity of 1,25(OH)_2_D_3_^9,17^. Thus, while 1,25(OH)_2_D_3_ shows a substantial calcemic effect at a low dose of 0.1 µg/kg^12^, 20*S*(OH)D_3_ does not cause hypercalcemia in rats^12^ and mice at doses as high as 60 µg/kg^22^. These results suggest that 20*S*(OH)D_3_ is a promising lead compound for the development of anti-inflammatory agents that lack toxic hypercalcemic effects at pharmacological doses.

In this study, a series of 20*S*(OH)D_3_ analogs with modified side chains were chemically synthesized. Modifications made were aimed to reduce or maintain a low rate of metabolism by CYP24A1, as displayed by the parent compound, and to potentially enhance the interaction with the VDR. The modifications included replacing C24 with an oxygen (**13**), inserting a double bond between C24 and C25 (**4**), adding an amide group replacing carbons 23 and 24 (**33**) and adding two fluorine groups at C24 (**23**). Their abilities to be 1α-hydroxylated by the activation enzyme, CYP27B1, and metabolized by the catabolic enzyme, CYP24A1, were evaluated. Two analogs (**4** and **33**) were chosen to co-crystalize with the VDR to decipher their differential modes of interaction in comparison with 20*S*(OH)D_3_ and 1,25(OH)2D_3_. The enzymatically-generated 1α-OH derivatives showed a much stronger ability to activate the VDR for the selected activities tested, including vitamin D response element (VDRE)-reporter (luciferase) activity, and CYP24A1 expression measured by real-time PCR, as compared to the parent compounds. Their abilities to inhibit the production of IFNγ by activated murine lymphocytes were also determined.

## Results and Discussion

### Synthesis of 20*S*(OH)D_3_ analogs and their 1α-OH derivatives

To investigate the effects of side-chain modification of VDR interaction and minimize metabolism by CYP24A1, a series of 20*S*(OH)D_3_ analogs were synthesized. In addition, to determine the influence of having the 1α-OH present, their 1α-OH derivatives were biosynthetically made using purified CYP27B1. The chemical procedures and details are listed in the Supplementary information.

**Figure 2:**
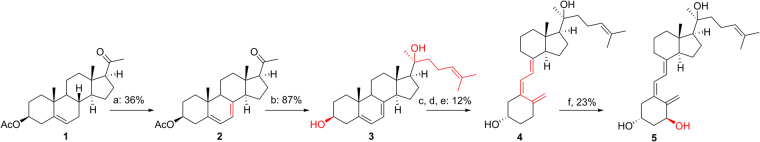
Synthesis of 20*S*(OH)D_3_ analog **4** and its 1α-OH derivative **5**. Reagents and conditions: (**a**) dibromantin, AIBN, benzene: hexane (1:1), reflux 20 min; TBAB, THF, r.t., 75 min, then TBAF, r.t., 50 min. (**b**) 5-Bromo-2-methyl-2-pentene, Mg, THF, 1 h; THF, 0 °C - r.t., 8 h. (**c**) UVB, Et_2_O, 15 min. (**d**) EtOH, reflux, 3 h. (**e**) HPLC, MeCN:H_2_O. (**f**) CYP27B1.

The synthesis of the first analog is shown in Fig. 2. We started from pregnenolone acetate **1** which was transformed into the 7-dehydrocholesterol (7DHC) type intermediate **2** by a well-established procedure with a 36% yield^1,4,23^. Grignard reaction using a self-made Grignard reagent removed the 3-acetyl, generated 20*S*-OH and added the modified side chain in one step to produce **3** with a satisfactory yield (87%). To open the B-ring to produce the D_3_-like structure, **3** dissolved in ethyl ether was irradiated with UVB light, followed by heat-induced isomerization to give the D_3_-like product **4** bearing a 24-ene with a 12% yield. Preparative HPLC was used to purify **4** from the reaction mixture using acetonitrile (MeCN) and water as mobile phases. The 1α-OH derivative **5** was produced by CYP27B1-mediated enzymatic catalysis which specifically adds the 1α-OH to the D_3_-like structures based on its function^24^, and was purified by HPLC.

**Figure 3:**
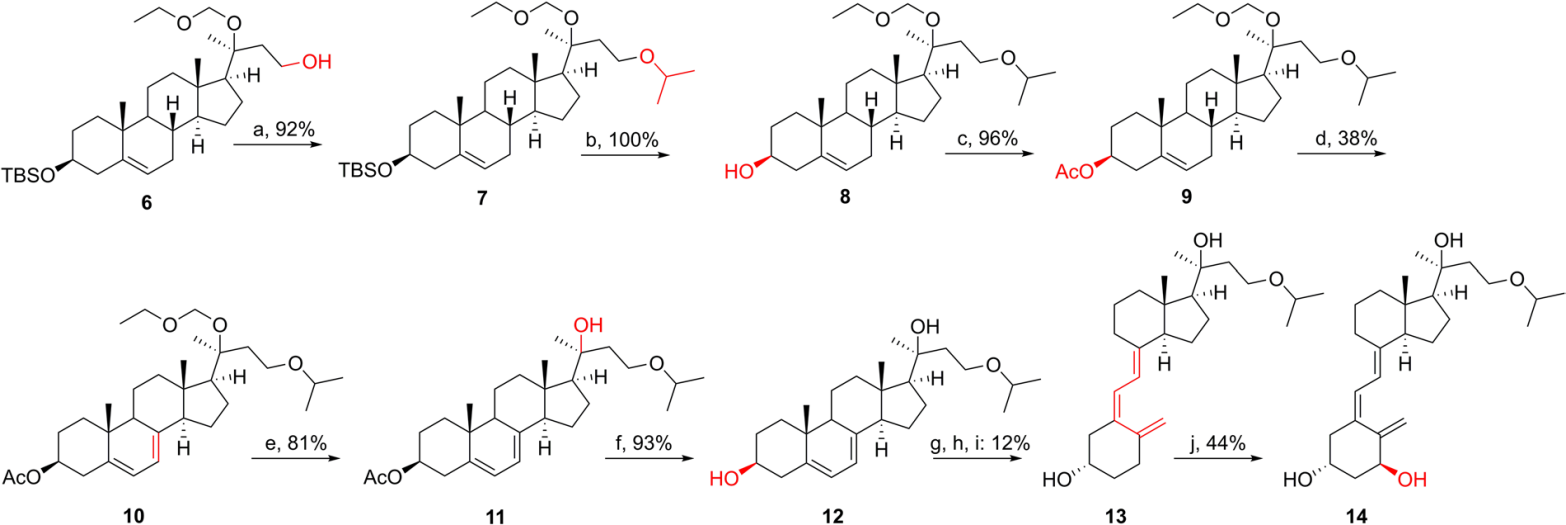
Synthesis of 20*S*(OH)D_3_ analog **13** and its 1α-OH derivative **14**. Reagents and conditions: (**a**) isopropyl bromine, NaH, THF, r.t., overnight. (**b**) TBAF, THF, r.t., 12 h. (**c**) Ac_2_O, pyridine, DMAP, 6 h. (**d**) dibromantin, AIBN, benzene: hexane (1:1), reflux 20 min; TBAB, THF, r.t., 75 min, then TBAF, r.t., 50 min. (**e**) CSA, MeOH:DCM (1:1), 0 °C - r.t., 12 h. (**f**) aq. KOH, MeOH, 2 h. (**g**) UVB, Et_2_O, 15 min. (**h**) EtOH, reflux, 3 h. (**i**) HPLC, MeCN:H_2_O. (**j**) CYP27B1.

The synthetic route for **13** and **14** bearing a 24-oxa group starting from **6**^1^ is shown in Fig. 3. Coupling of isopropyl bromine and **6** under basic condition gave **7** with a 92% yield. After replacing 3-OTBS with 3-OAc to afford **9**, the 7DHC-like intermediate (**10**) was produced by the above-mentioned procedure (38% yield). EOM deprotection (81%) and ester hydrolysis (93%) were carried out under acidic condition (CSA) and basic condition (KOH), separately, to afford the final 7DHC intermediate **12**. Similarly, the D_3_ structure **13** and its 1α-OH derivative **14** were obtained from the B-ring opening reaction and enzymatic transformation, respectively.

**Figure 4:**
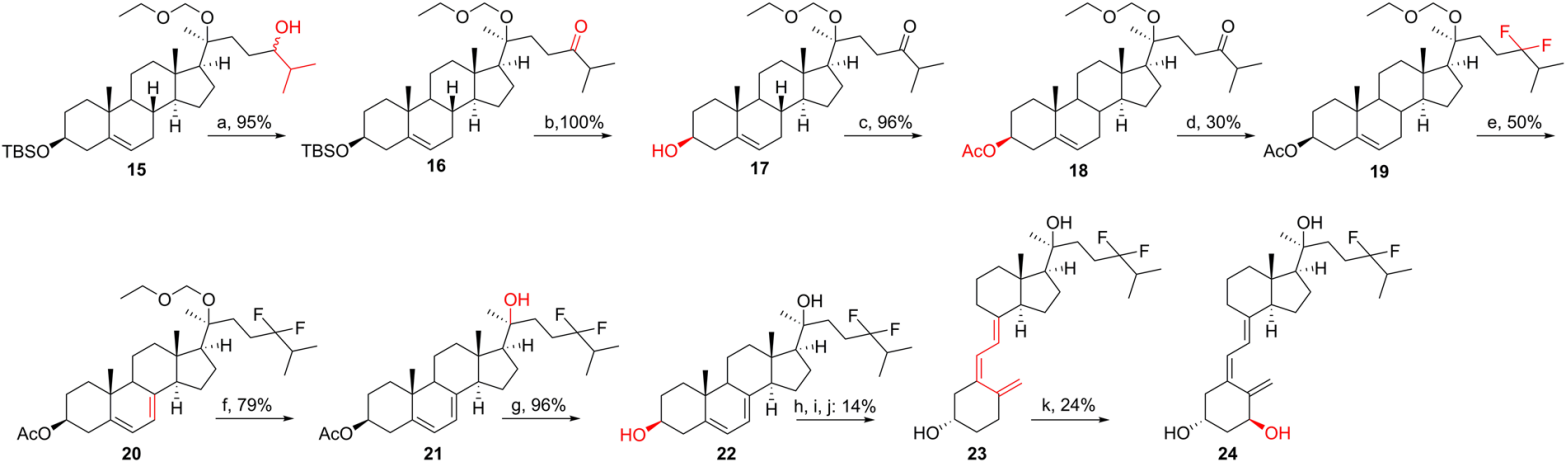
Synthesis of 20*S*(OH)D_3_ analog **23** and its 1α-OH derivative **24**. Reagents and conditions: (**a**) PDC, CH_2_Cl_2_, r.t., 24 h. (**b**) TBAF, THF, r.t., 12 h. (**c**) Ac_2_O, pyridine, DMAP, 6 h. (**d**) DAST, DCM, r.t. −40 °C, 48 h. (**e**) Dibromantin, AIBN, benzene: hexane (1:1), reflux 20 min; TBAB, THF, r.t., 75 min, then TBAF, r.t., 50 min. (**f**) CSA, MeOH:DCM (1:1), 0 °C - r.t., 12 h. (**g**) aq. KOH, MeOH, 2 h. (**h**) UVB, Et_2_O, 15 min. (**i**) EtOH, reflux, 3 h. (**j**) HPLC, MeCN:H_2_O. (**k**) CYP27B1.

The synthetic route for **23** and **24** bearing a 24-difluoro modification started from **15**^23^ (Fig. 4). PDC oxidation of **15** gave ketone **16** with 95% yield, which was followed by replacing the 3-OTBS with 3-OAc to afford **18** which underwent DAST fluorination to produce **19** with a 30% yield. The 7DHC-like intermediate (**20**) was produced by the above-mentioned procedure (50% yield), then underwent EOM deprotection (79%) and ester hydrolysis (96%) under acidic condition (CSA) and basic condition (KOH), separately, to afford the final 7DHC-like intermediate **22**. Similarly, the D_3_-like structure **23** and its 1α-OH derivative **24** were obtained from the B-ring opening reaction and enzymatic transformation, respectively.

**Figure 5:**
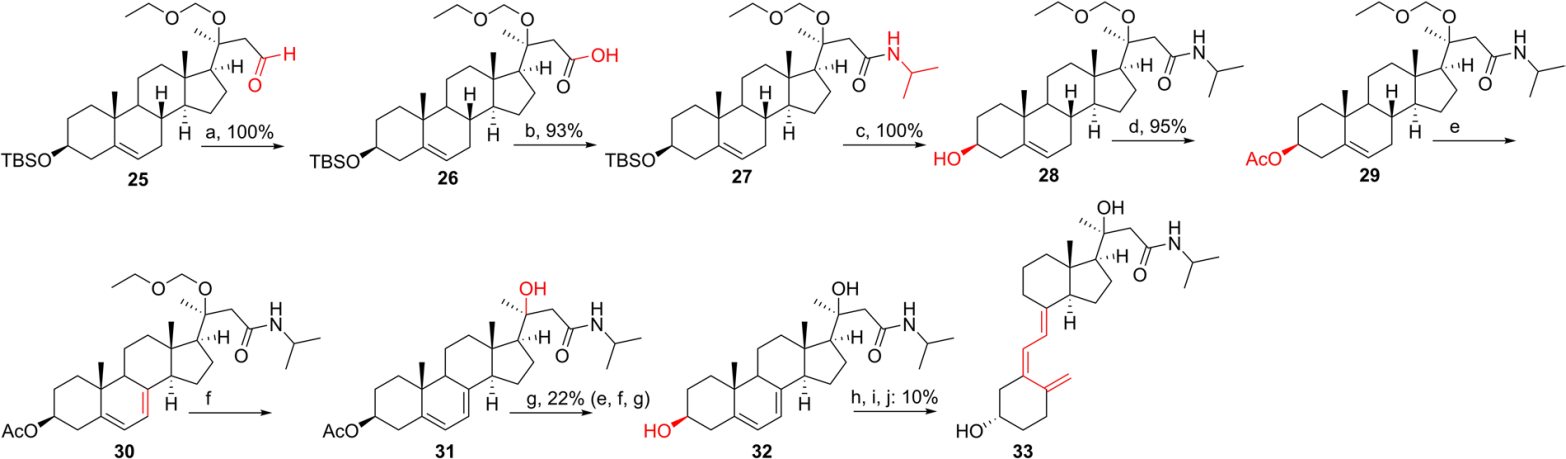
Synthesis of 20*S*(OH)D_3_ analog **33**. Reagents and conditions: (**a**) NaIO_4_, NaH_2_PO_4_, H_2_O_2_, MeCN:THF (10:1), 0 °C - r.t., 2 h. (**b**) PyBOP, isopropylamine, pyridine, 0 °C - r.t., 12 h. (**c**) TBAF, THF, r.t., 12 h. (**d**) Ac_2_O, Et_3_N, DMAP, DCM, 12 h. (**e**) Dibromantin, AIBN, benzene: hexane (1:1), reflux 20 min; TBAB, THF, r.t., 75 min, then TBAF, r.t., 50 min. (**f**) CSA, MeOH:DCM (1:1), 0 °C - r.t., 12 h. (**g**) aq. K_2_CO_3_, MeOH, 12 h; HPLC. (**h**) UVB, Et_2_O, 15 min. (**i**) EtOH, reflux, 3 h. (**j**) HPLC, MeCN:H_2_O.

The synthetic route for **33** using intermediate **25**^1^ is shown in Fig. 5. According to a previous procedure^25^, **25** was oxidized into acid **26** under mild condition without affecting the acid-sensitive TBS and EOM protection. Amide coupling of **26** and isopropylamine to construct the side chain were catalyzed by PyBOP with a 93% yield. After replacing 3-OTBS with 3-OAc to afford **29**, the final 7DHC-like intermediate (**32**) was produced by the above-mentioned dibromatin/AIBN/TBAB/TBAF reaction, then EOM deprotection and ester hydrolysis, with a 22% yield for the three steps. Similarly, the D_3_-like structure **33** having a 23-amide modification was obtained from the the photochemical opening of the B-ring. Its 1α-OH derivative, however, could not be generated by CYP27B1 (discussed later).

The synthesis of analog **4** is relatively simple due to the commercially available reagent 5-bromo-2-methyl-2-pentene, making the side chain completion possible via a one-step Grignard reaction. In contrast, we have to construct the modified side chains for analogs **13**, **23** and **33** by multi-step schemes, and the previously made intermediates (**6**, **15** and **25**) have greatly sped up the synthesis of these analogs. The chemical addition of 1α-OH is not straightforward, thus the well-established biosynthetic strategy using CYP27B1 was applied here to produce sufficient of the 1α-hydroxy-derivatives for biological testing. Recently, we have worked out a synthetic route for producing 1α,20*S*(OH)_2_D_3_ chemically^26^, the same strategy can be also applied to the chemical synthesis of the 1α-OH derivatives in the future.

### Metabolism profiles of 20*S*(OH)D_3_ analogs by CYP24A1

Since CYP24A1 is the major enzyme determining the metabolic stability (half-life) of 20*S*(OH)D_3_ and 1,25(OH)_2_D_3_, we tested its ability to metabolize the 20*S*(OH)D_3_ analogs. Fig. S1 shows HPLC analyses of the products resulting from a 10 min incubation of 20*S* (OH)D_3_, **4**, **13**, **23** and **33** with rat CYP24A1 and reveals that this enzyme is capable of metabolizing all these compounds, despite the structural differences. Three major products were observed with 20*S*(OH)D_3_ as substrate (Fig. S1B) that were not present in the control (Fig. S1A), similar to what was reported before^27,28^. These were identified from authentic standards as 20*S*,25(OH)_2_D_3_, and the two C24 diastereomers of 20*S*,24(OH)_2_D_3_^23,27^. One of the C24 diastereomers, 20*S*,24 *R*(OH)_2_D_3_ is the major product and accounts for almost 70% of the total products. The action of CYP24A1 on **4** resulted in at least nine products (Fig. S1D), suggesting a complex pathway of metabolism. There were two major products formed from this analog, RT27 and RT30 (indicated by arrows), which accounted for approximately 20% and 30% of the total products, respectively. Compound **23** (Fig. S1E) was converted to at least 6 different products by the incubation with CYP24A1. In contrast, **13** tested under identical conditions generated only one major product with a retention time of 20 min (RT20) and three minor products (Fig. S1D). By 10 min of incubation, almost all of **13** had been metabolized by CYP24A1. Three major products (indicated by arrows) and at least three minor ones were observed for the metabolism of **33** by CYP24A1 (Fig. S1F).

### Time courses for the metabolism of the 20*S*(OH)D_3_ analogs by CYP24A1

**Figure 6:**
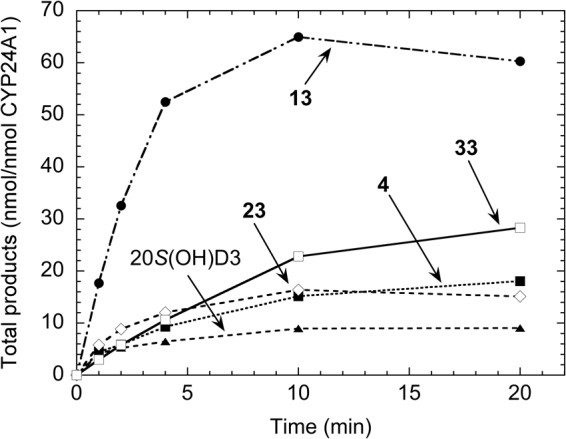
Time courses for metabolism of 20*S*(OH)D_3_ analogs in phospholipid vesicles by rat CYP24A1. 20*S*(OH)D_3_, **4**, **13**, **23** and **33** were incorporated into phospholipid vesicles at a ratio of 0.018 mol/mol phospholipid and incubated with 0.14 µM rat CYP24A1 at 37 °C. Products were analyzed by HPLC.

The activity of CYP24A1 with the different analogs was determined as a function of time to determine the initial linear region that could be used for kinetic studies, and to determine any differences in the pattern between analogs. These time courses revealed that by 20 min there was 6-fold greater metabolism of **13** than for 20*S*(OH)D_3_ (Fig. 6). The major product of **13** (Fig. S1C, RT20), accounted for 71% of the total secosteroids (products and substrate) at one min and by 20 min it was 95% of all secosteroids. The time course for 20*S*(OH)D_3_ in comparison to **4** and **33**, reached maximum product formation early in the incubation, with the amount of product at the end of the incubation being only 2.2-fold higher than at one min. By 20 min, less than 15% of the 20*S*(OH)D_3_ had been metabolized. These data suggests that products of the metabolism of 20(OH)D_3_ by CYP24A1 may inhibit the enzyme. While initially being metabolized at the lowest rate of any of the analogs, **33** maintained its initial rate longer with more product being present at the end of the 20 min incubation than for any of the other analogs except **13** (Fig. 6).

### Kinetics of the metabolism of 20*S*(OH)D_3_ analogs by CYP24A1

To more fully document the differences in catalytic activities between the analogs revealed by Fig. 6, the kinetic parameters of CYP24A1 for the metabolism of the 20-hydroxy analogues were determined. An incubation time of 1 min was chosen to obtain the initial rate based on the time courses (Fig. 6) and previous studies^28,29^. 1,25(OH)_2_D_3_ was included for comparison and all analogs displayed higher K_m_ values than this major substrate for CYP24A1, reflecting their lower affinity for the active site of the enzyme. Only **13** displayed a higher maximum catalytic activity than 1,25(OH)_2_D_3_ under saturating substrate conditions (k_cat_), with 4 and 33 displaying the lowest k_cat_ values which were approximately 4-fold lower than for 1,25(OH)_2_D_3_. K_m_ values varied greatly between the 20*S*(OH)D_3_ analogs (Table 1). **4** had the lowest K_m_ for CYP24A1, whereas 20*S*(OH)D_3_ displayed the highest which was over 17-fold higher than for **4**. **13** and **23** displayed similar K_m_ values, 3–4 fold lower than that for 20*S*(OH)D_3_, while **33** gave a K_m_ half that of 20*S*(OH)D_3_. The resulting k_cat_/K_m_ values which give a measure of catalytic efficiency under conditions of low substrate concentration, show that **13** is most efficiently metabolized by rat CYP24A1 with a value 72% higher than for the next best analog, **4**. 20*S*(OH)D_3_ and **33** displayed similar catalytic efficiencies which were the lowest of the analogs tested and 13-fold lower than for **13**. Thus, the modifications at C24 that were introduced generally increased the efficiency of their metabolism. This can be explained in part by the presence of additional functional groups on the side chain increasing the interaction with CYP24A1 active site, thus lowering the K_m_. The known ability of CYP24A1 to hydroxylate the vitamin D side chain from C23 to C27 depending on what prior functional groups are present^27,29^ provides an explanation as to why blocking of one or two carbons on the side chain from hydroxylation does not prevent hydroxylation at neighboring carbons. It is important to note that all the new 20*S*(OH)D_3_ analogs were metabolized by CYP24A1 with a catalytic efficiency at least 5-fold lower than 1,25(OH)_2_D_3_ (Table 1), suggesting that they are likely to display increased metabolic stability *in vivo*.

**Table 1:** Kinetic data for the metabolism of the 20*S*(OH)D_3_ analogs by rat CYP24A1.

Substrate	K_m_ (mmol/mol PL)	k_cat_ (min^−1^)	k_cat_/K_m_ (mol PL.mmol^−1^.min^−1^)
**4**	2.0 ± 1.7	4.2 ± 0.7	2.16
**13**	7.9 ± 1.5	29.2 ± 1.5	3.72
**23**	11.6 ± 3.1	7.8 ± 0.6	0.67
**33**	15.4 ± 1.7	4.8 ± 0.2	0.31
20*S*(OH)D_3_	34.8 ± 9.4	10.1 ± 1.2	0.29
1,25(OH)_2_D_3_	0.98 ± 0.30	19.7 ± 0.7	20.1

Analogs were incorporated into phospholipid vesicles and incubated for one min in a reconstituted system with rat CYP24A1 (0.14 µM) and a range of substrate concentrations. Kinetic parameters were determined by fitting hyperbolic curves to the data using KaleidaGraph version 4.1. Data for K_m_ and k_cat_ are shown ± SE from the curve fit. PL: phospholipid.

### Metabolism of 20S(OH)D_3_ analogs by CYP27B1

In order to make the 1α-OH derivatives of the 20*S*(OH)D_3_ analogs enzymatically, and to assess their likely capacity to be 1α-hydroxylated *in vivo*, the ability of mouse CYP27B1 to hydroxylate these metabolites was examined. 25(OH)D_3_, the major natural substrate for CYP27B1^2,30^, was included for comparison. The 20*S*(OH)D_3_ analogs were incorporated into phospholipid vesicles and incubated with CYP27B1, then the extent of their metabolism was determined by HPLC (Table 2). All analogs tested except **33** were metabolized by CYP27B1. In each case only a single metabolite was produced, assumed to be the 1α-OH derivative based on the known high specificity of the enzyme for the 1α-position^24,30,31^. Analog **13** showed the highest conversion to product in the 20 min incubation, with greater than 90% conversion which was slightly higher than for 25(OH)D_3_ and 4.3 fold higher than that observed with 20*S*(OH)D_3_. Metabolism of **4** was almost twice that of 20*S*(OH)D_3_ and metabolism of **23** almost three fold. Thus, the modifications to C24 and/or C25 seen in **13**, **4** and **23** enhanced their ability to be metabolized by CYP27B1. The replacement of carbon 24 with an oxygen atom had a similar effect to adding a hydroxyl group at C24 which we previously showed increased the catalytic efficiency of 1α-hydroxylation by both increasing the k_cat_ and decreasing the K_m_ relative to the values seen for 20*S*(OH)D_3_^31^. In contrast, the introduction of an amide linkage into the side chain, as in **33**, prevented its hydroxylation by CYP27B1. In a separate experiment we found that when present with an equal concentration 25(OH)D_3_, **33** was able to reduce the metabolism of 25(OH)D_3_ from 82% to 56% in a 20 min incubation. This suggests that it is a competitive inhibitor and can compete for binding to the active site of CYP27B1 with 25(OH)D_3_, but binds in an unfavorable position for hydroxylation.

**Table 2:** Metabolism of the 20*S*(OH)D_3_ analogs by mouse CYP27B1.

Substrate	Product (% total secosteroids)
**4**	40.6
**13**	91.3
**23**	57.1
**33**	0
20*S*(OH)D_3_	21.1
25(OH)D_3_	86.0
25(OH)D_3_ + **33** (1:1)	53.0

20*S*(OH)D_3_ analogs were incorporated into phospholipid vesicles at a ratio of 0.018 mol/mol phospholipid and incubated with 0.8 µM mouse CYP27B1 for 20 min at 37 °C. Products were analyzed by reverse phase HPLC using an acetonitrile in water gradient (see Methods).

### VDRE stimulation activity

Using our previously established VDRE-Luciferase reporter models^1,4,23,32^, the VDR-induced transcriptional activity of the analogs and their 1α-OH derivatives were investigated in three different cell lines (Caco-2, HaCaT and Jurkat). As shown in Table 3, both positive controls, 1,25(OH)_2_D_3_ and 22-Oxa, showed strong activity for VDR activation in all three cell lines, with 22-Oxa being the most potent one among all compounds tested. 1,25(OH)_2_D_3_ and 22-Oxa showed better activities in Jurkat cells than in Caco-2 or HaCaT cells, suggesting that they are more sensitive to immune cells. 20*S*(OH)D_3_ analogs were unable to significantly activate VDR except **4** which showed moderate activity. In Caco-2 and HaCaT cells, all 1α-OH derivatives displayed potent activities which were better than or comparable with that of 1,25(OH)_2_D_3_, the native ligand of VDR, suggesting that these analogs were also strong VDR agonists. The activities of 1α-OH derivatives in Jurkat cells were as potent as in Caco-2 and HaCaT cells. The better activities of 1α-OH derivatives over their parent analogs suggested the importance of 1α-OH for VDR activation with the synthetic VDRE used in these assays, which is consistent with our previous studies on 20*S*,23 *S*/*R*(OH)_2_D_3_^1^ and 20*S*,24 *S*/*R*(OH)_2_D_3_^23^. One thing noteworthy is that **4** and **5** had relatively better activities than the other analogs and stood out amongst the 20*S*(OH)D_3_ analogs and their 1α-OH derivatives, respectively. It would therefore appear that the presence of the 24-ene group inside the VDR binding pocket enhances VDR activation, particularly when the 1α-OH is also present.

**Table 3:** VDRE stimulation and anti-inflammatory activities of 20*S*(OH)D_3_ analogs and their 1α-OH derivatives.

Compound	VDRE stimulation (EC_50_ ± SD, nM)			IFNγ (ratio ± SD)
	Caco-2	HaCaT	Jurkat	
**4**	580.2 ± 19.2	460.4 ± 36.5	439.3 ± 25.7	0.832 ± 0.023^*^
**13**	NS	NS	NS	0.824 ± 0.031^**^
**23**	NS	NS	NS	0.698 ± 0.031^***^
**33**	NS	NS	NS	0.628 ± 0.014^***^
**5**	181.6 ± 6.1	197.8 ± 8.1	237.4 ± 5.5	0.381 ± 0.017^***^
**14**	188.0 ± 1.9	226.5 ± 3.5	248.4 ± 5.0	0.628 ± 0.008^***^
**24**	235.7 ± 4.1	254.1 ± 7.9	305.0 ± 4.5	0.560 ± 0.010^***^
20*S*(OH)D_3_	NS	NS	NS	0.517 ± 0.046^***^
1,25(OH)2D_3_	465.3 ± 20.9	305.2 ± 12.4	31.8 ± 4.5	0.397 ± 0.049^***^
22-Oxa	42.5 ± 1.6	40.1 ± 1.5	2.9 ± 0.3	0.487 ± 0.017^***^
Control	NA	NA	NA	1.000 ± 0.015

SD: standard deviation, NS: no significance, NA: not applicable. *p < 0.05, **p < 0.01 and ***p < 0.001 compared with the control.

### RT-PCR-based analysis of *CYP24A1* expression

**Figure 7:**
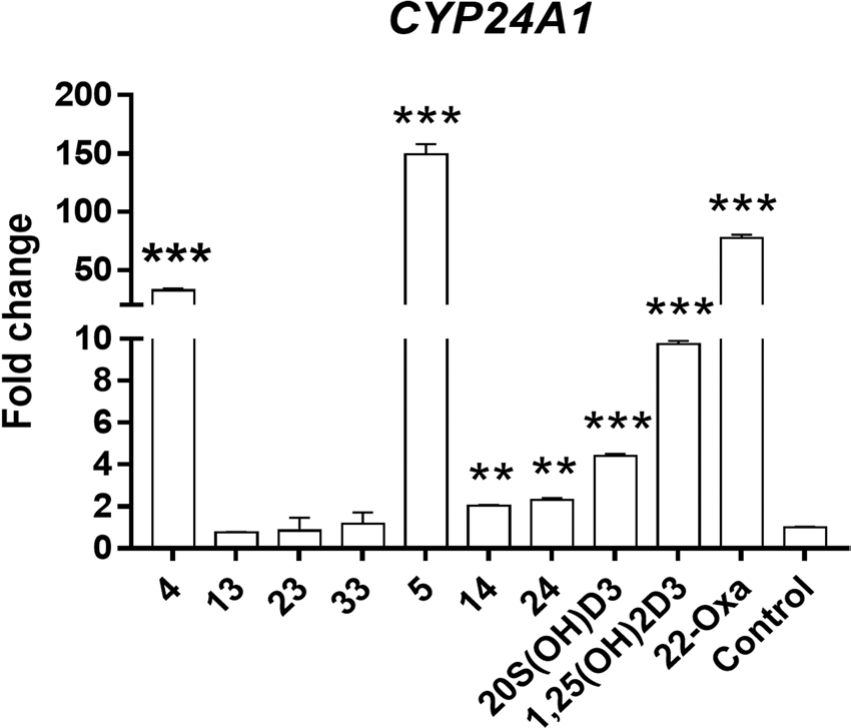
20*S*(OH)D_3_ analogs upregulate the expression of *CYP24A1* encoding the vitamin D catabolic enzyme, CYP24A1, with varying efficiencies. HaCaT cells were treated with 100 nM concentrations of analogs, 20*S*(OH)D_3_, 1,25(OH)_2_D_3_ or 22-Oxa, or with DMSO only (vehicle) as a control. The mRNA was isolated and the RT-PCR was performed using specific primers for the *CYP24A1* gene (see Methods). Data are presented as mean ± SE (*n* = 3). *p < 0.05, **p < 0.01 and ***p < 0.001 compared with the control.

We have previously shown that 20*S*(OH)D_3_ causes poor stimulation of CYP24A1 expression compared to 1,25(OH)2D_3_^32,33^, despite having comparable potency for inhibiting keratinocyte proliferation^11,33^. We therefore compared the activity of the five synthetic analogs on the expression of the *CYP24A1* gene in HaCaT cells to that of 1,25(OH)_2_D_3_ and 22-Oxa. As shown in Fig. 7, after 24 h treatment with 100 nM of each analog, relative mRNA levels for *CYP24A1* were 32.4-, 0.8-, 0.9-, and 1.2-fold higher relative to the negative control for analogs **4**, **13**, **23** and **33**, respectively, suggesting that analog **4** was strongly upregulating VDR target gene expression via the VDR while the other analogs lacked such activity. After introduction of the 1α-OH group, the ability to stimulate *CYP24A1* expression was significantly improved as illustrated by the results for **5**, **14** and **24**. They showed mRNA levels for *CYP24A1* at 149.1-, 2.0- and 2.3-fold that of the vehicle-control. In comparison, cells treated with 20*S*(OH)D_3_, 1,25(OH)_2_D_3_ or 22-Oxa at the same concentration showed a 4.4-, 9.8- or 77.5-fold increase in mRNA for *CYP24A1*, respectively. These results suggested that both 24-ene and 1α-OH modifications enhanced the ability of the secosteroids with a 20*S*-OH group to activate the VDR when associated with the VDRE-elements (Table 3) in the *CYP24A1* gene.

### Inhibitory activity on IFNγ production

1,25(OH)_2_D_3_ and some of its analogs can act as immunomodulatory agents and have anti-inflammatory activities^1,34^. IFNγ is an important cytokine of the immune system and is a common inflammation marker. Our previous studies have demonstrated that both 1,25(OH)_2_D_3_ and 20*S*(OH)D_3_ significantly inhibit IFNγ production with similar potency^9^. To test whether the 20*S*(OH)D_3_ analogs also show anti-inflammatory properties, the inhibition of IFNγ production was performed using our established assay^1,4,23^. As shown in Table 3, positive controls 1,25(OH)2D_3_, 22-Oxa and 20*S*(OH)D_3_ showed similar effects on IFNγ production, decreasing its level by 60%, 51% and 48% at 100 nM, respectively. All chemically synthesized 20*S*(OH)D_3_ analogs significantly reduced IFNγ concentrations, however, to a lesser degree than 1,25(OH)2D_3_, 22-Oxa and 20*S*(OH)D_3_. After 1α-hydroxylation, the inhibitory activities of all 1α-OH derivatives (37% to 62% decreases) were significantly improved as compared with their parent analogs (16.7% to 30% decreases). Among these compounds, **5** was the most active one because it showed comparable reduction of IFNγ production to 1,25(OH)_2_D_3_ and larger reduction than 22-Oxa (*p* < 0.001). These results suggest that 1α-hydroxylation of these 20*S*(OH)D_3_ analogs may improve their anti-inflammatory activity, which may also be affected by specific side chain modifications.

### Crystal structures of 20*S*(OH)D_3_ analogs in complex with the zebrafish VDR ligand binding domain

**Figure 8:**
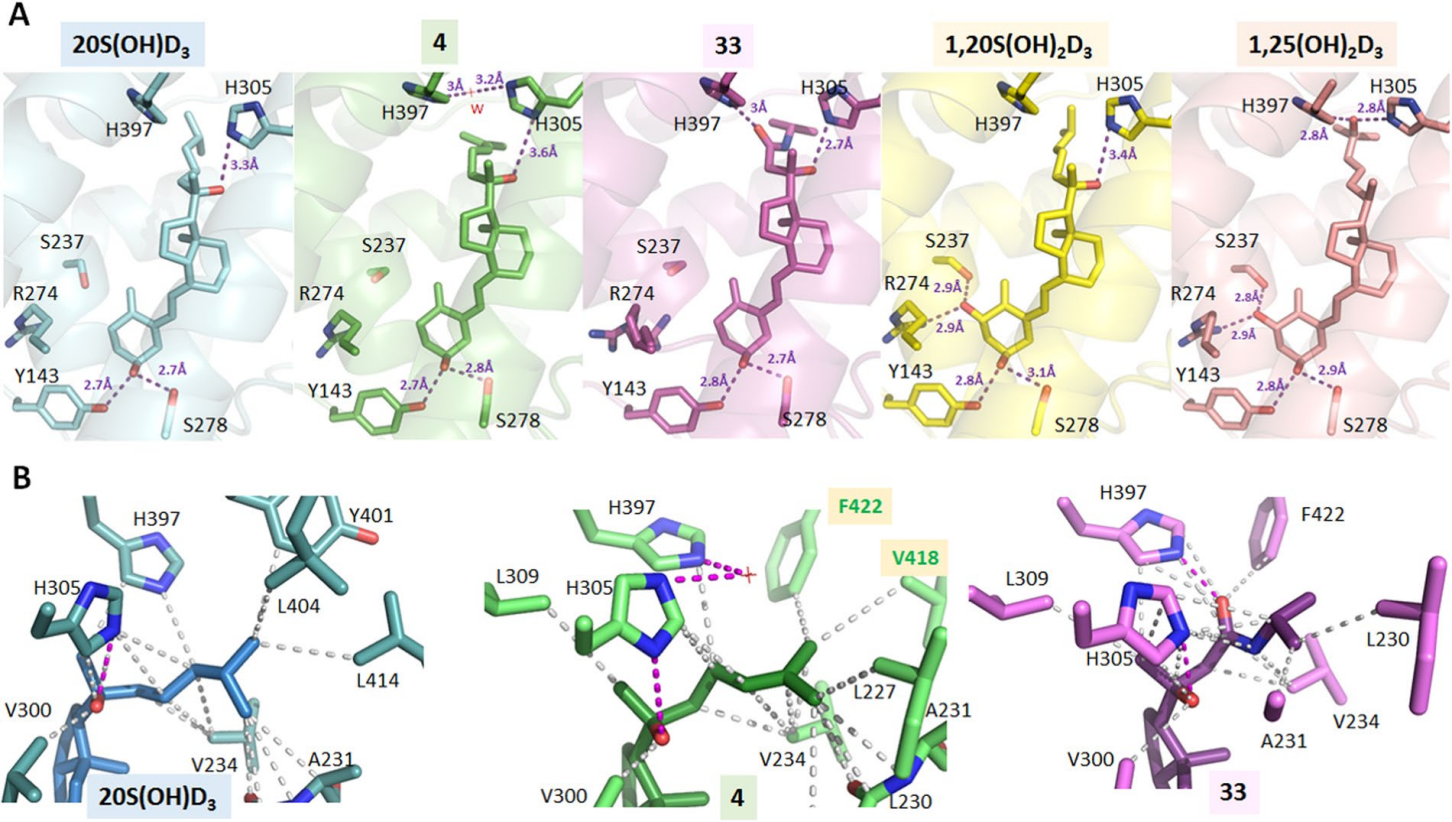
Detailed views of 20*S*(OH)D_3_ analogs/VDR LBD complexes. (**A**) Hydrogen-bonding network between 20*S*(OH)D_3_ analogs and zVDR LBD/SRC1 complexes. The complexes with 20*S*(OH)D_3_ (PDB ID 5OW9), **4** (PDB ID 5OWD), **33** (PDB ID 5OW7), 1,20*S*(OH)_2_D_3_ (PDB ID 5MX7) and 1,25(OH)_2_D_3_ (PBD ID 2HC4) are shown in cyan, green, pink, yellow, and salmon, respectively. Hydrogen bonds between the ligands and LBD are represented by purple dashed lines. A dual conformation of the side chain of Arg274 is observed in the VDR/**33** complex. (**B**) Details of the interactions mediated by the side chains of 20*S*(OH)D_3_ analogs. Hydrophobic interactions are indicated by grey dashed lines, and hydrogen bonds are depicted as pink dashed lines. Only residues within 4 Å of the ligand are shown by stick representation. A water molecule (W) depicted by a red cross interacts with His397 and His305 in the VDR/**4** complex. The residues numbers correspond to human VDR.

We previously characterized the binding mode of 1,20*S*(OH)_2_D_3_ to the VDR ligand binding domain (LBD), showing that the 20*S*-OH group forms a weak H-bond with His305^26^. The crystal structures of 20*S*(OH)D_3_, **4** and **33** analogs in complex with the *Danio Rerio* VDR (zVDR) LBD were determined and compared to those of 1,20*S*(OH)_2_D_3_ and 1,25(OH)_2_D_3_ VDR complexes (Fig. 8). 20*S*(OH)D_3_ interacts similarly to 1,20*S*(OH)_2_D_3_ but lacks the H-bonds with Ser237 and Arg302 (Fig. 8A). The interaction of the 20*S*-OH with H305 in **4** is similar to that for 1,20*S*(OH)_2_D_3_ forming a weak H-bond. Due to the 24-ene group in **4**, the terminal methyl groups are differentially positioned forming stronger contacts with C-terminal residues, Val418 and Phe422, compared to 20*S*(OH)D_3_, that stabilize the agonist conformation of VDR in agreement with the strongest transcriptional activity of analog **4** amongst the 20*S*(OH)D3 analogs (Fig. 8B). Analogs **4** and **33** cause conformational changes in the position of His305 and His397 (Fig. S2) with quite a substantial change in the case of **33** which formed a hydrogen bond between His397 and the amide group which is in contrast to 20*S*(OH)D_3_ which does not interact with this residue. While the exact relationship between these conformational changes induced by **33** and VDR function remain to be elucidated, the change indicates the possibility of producing analogs that display a unique subset of the actions seen for 1,25(OH)_2_D_3_ (i.e. are biased agonists), as already seen to some degree for the parental 20*S*(OH)D_3_. They also substantiate previous molecular modeling studies that showed high docking scores for 20*S*(OH)D_3_ and functional assays showing its ability to efficiently translocate VDR from the cytoplasm to the nucleus^9,15,17,18^.

## Conclusion

In this study, four novel 20*S*(OH)D_3_ analogs with side chain modifications were chemically synthesized, and their 1α-OH derivatives were produced enzymatically using recombinant CYP27B1. Enzymatic studies showed that CYP27B1 can hydroxylate the novel 20*S*(OH)D_3_ analogs at C1α (except **33**). All analogs served as substrates for CYP24A1 despite the modifications made at C24, but were much more metabolically stable than 1,25(OH)_2_D_3_. The 20*S*(OH)D_3_ analogs showed VDR-stimulatory and VDR-downstream-gene regulatory activities that were significantly improved by 1α-hydroxylation. In addition, 1α-hydroxylation improved the inhibition of IFNγ production by activated lymphocytes. Co-crystal structures of VDR in complex with 20*S*(OH)D_3_, **4**, and **33** revealed notable differences in their molecular interactions in the binding pocket of VDR, which will be insightful for developing novel VDR agonists as anti-inflammatory agents.

## Methods

### General methods

All reagents and solvents in synthetic and separation procedures were purchased from commercial sources and were used as received unless otherwise noted. Reactions of 5,7-diene structures were all protected by wrapping the flasks with aluminum foil. Moisture- or oxygen-sensitive reactions were performed under an argon atmosphere. All reactions were routinely monitored by TLC on silica gel, and visualized by 5% phosphomolybdic acid in ethanol for non-UV active compounds or UV lights for compounds with absorption at 254 nm. Ethyl acetate was used for extraction of reaction mixtures and then dried over anhydrous Na_2_SO_4_, filtered and removed using a rotary evaporator under reduced pressure. Mass spectra of all compounds were obtained using a Bruker ESQUIRE-LC/MS system equipped with an ESI source (Billerica, MA, USA). The purities of final D_3_ compounds, as analyzed by an Agilent 1100 HPLC system (Santa Clara, CA, USA), were above 98%. High-resolution MS spectra were obtained from a Waters UPLC-Q/Tof-MS system with the function of molecular formula prediction (Milford, MA, USA). NMR data were collected at 25 °C. Chemical shifts were referenced to residual solvent peaks of methanol-*d*_4_ or CDCl_3_. NMR measurements were performed on either a Bruker Avance III 400 MHz (Bruker BioSpin, Billerica, MA, USA), or a Varian Unity Inova 500 MHz spectrometer (Agilent Technologies Inc., Santa Clara, CA, USA).

### Metabolism of analogs by CYP24A1 and CYP27B1

Rat CYP24A1, mouse CYP27B1 and adrenodoxin and human adrenodoxin reductase were expressed in *E. coli* and purified as described before^28,30,35^. To test metabolism of each analog, they were incorporated into phospholipid vesicles made from dioleoyl phosphatidylcholine and cardiolipin by sonication, as before^24,28^. The substrates in vesicles (510 µM phospholipid) were incubated at 37 °C with either CYP24A1 (0.14 µM) or CYP27B1 (0.8 µM) in a reconstituted system containing adrenodoxin (15 µM) and adrenodoxin reductase (0.4 µM). Samples from incubations with CYP24A1 were extracted with dichloromethane and analysed by HPLC using a 25 cm Grace Alltima C18 column, as before^27,28^. Products (except **33**) were separated using an acetonitrile on water gradient (45% to 100% for 20 min then 100% acetonitrile for 40 min at a flow rate of 0.5 mL/min). For the more polar **33**, the acetonitrile gradient was 30% to 100% acetonitrile for 30 min then 100% acetonitrile for 20 min, at 0.5 mL/min. Products from incubations with CYP27B1 were similarly extracted with dichloromethane and analyzed by reverse phase HPLC using a 15 cm Grace Smart C18 column and an acetonitrile in water gradient (10 min 45% to 100% acetonitrile then 20 min at 100% acetonitrile, at 0.5 mL/min).

### VDRE reporter assays

Caco-2, HaCaT and Jurkat cells were cultured as described previously^1,4,23,32^, and were transduced with lentiviral VDRE luciferase using a Cignal Lenti VDRE Reporter (luc) Kit according to the manufacturer’s protocol (QIAGEN, Valencia, CA, USA). After one week selection by puromycin (1 µg/mL), cells were seeded in a 96-well plate (10,000 cells/well with a volume of 100 µL/well) using FBS-free medium and synchronized for 24 h. DMSO solutions (1 µL) of secosteroids to be tested were added to cells, which were then incubated for another 24 h. The luciferase signal was then measured according to the manufacturer’s procedure for the ONE-Glo^TM^ Luciferase Assay System (Promega, Madison, WI, USA). The final concentration of DMSO was 0.1% and 0.1% DMSO was used as the vehicle control. All concentrations were tested in triplicate.

### Real time PCR-based gene expression analysis

HaCaT cells were purchased from Thermo Fisher Scientific (Waltham, MA, USA) and were cultured as for the VDRE reporter assay. The RNA from HaCaT keratinocytes treated with secosteroids or from DMSO controls was isolated using the Absolutely RNA Miniprep Kit (Stratagene, La Jolla, CA, USA). Reverse transcription (100 ng RNA/reaction) was performed with the Transcriptor First Strand cDNA Synthesis Kit (Roche Inc., Mannheim, Germany). Real-time PCR was performed using cDNA diluted 10-fold in sterile water and a SYBR Green PCR Master Mix. The primers for both forward and reverse strands for CYP24A1 were designed based on the mouse and rat sequences using Primer Quest software (Integrated Device Technology, San Jose, CA, USA). Reactions (in triplicate) were performed at 50 °C for 2 min, 95 °C for 10 min and then 40 cycles of 95 °C for 15 s, 60 °C for 30 s and 72 °C for 30 s. Data were collected and analyzed on a Roche Light Cycler 480. The amount of the final amplified product for each gene was compared and normalized to the amount of β-actin product as a housekeeping gene using a comparative Ct method^11^.

### IFNγ inhibition assay

Secosteroids were solubilized in absolute EtOH at 10^−4^ M and diluted to 10^−6^ M by adding Eagles Minimal Essential Medium (EMEM) containing 9% charcoal-stripped fetal calf serum, 100 U/mL penicillin and 100 µg/mL streptomycin, non-essential amino acids, 2.5 mM 2-mercaptoethanol, 2.5 mM L-glutamine^16^. Splenocytes from mice were isolated, erythrocytes lysed by hypotonic shock, washed twice with EMEM, and suspended at a concentration for 2 × 10^6^ cells/mL in EMEM as described above. To each well in a 48-well tissue culture plate, 450 µL of the splenocytes were added. Secosteroids (50 µL of the 10^−6^ M stock) or EtOH diluted 1:100 with the above culture medium were added to triplicate wells and then incubated at 37 °C in 5% CO_2_ in a humidified tissue culture incubator for 2 h, after which 1 µg/well of rat anti-mouse CD3 MOAB was added. After 72 h culture, supernatants from each well were harvested and analyzed by ELISA for levels of D-murine IFNγ (RAD Systems, Minneapolis, MN, USA), according to the manufacturer’s instructions. The concentration of IFNγ is supernatants from cultures containing secosteroids were compared to the concentration of IFNγ in the supernatants of EtOH-treated control cultures, by ANOVA.

### Co-crystallization of VDR in complex with secosteroids

zVDR LBD (156–453 AA) was produced and purified as previously described^36^. The protein was concentrated using an Amicon ultra-30 (Millipore) to 3–7 mg/mL and incubated with a two-fold excess of ligand and a three-fold excess of the coactivator SRC-1 peptide (686-RHKILHRLLQEGSPS-698). Crystals were obtained in 50 mM Bis-Tris pH 6.5, 1.6 M lithium sulfate and 50 mM magnesium sulfate. Protein crystals were mounted in a fiber loop and flash-cooled under a nitrogen flux after cryo-protection with 20% glycerol. Data collection from a single frozen crystal was performed at 100 K on the ID30 beamline at ESRF (France). The raw data were processed and scaled with the HKL2000 program suite^37^. The crystals belong to the space group P6522, with one LBD complex per asymmetric unit. The structure was solved and refined using BUSTER^38^, Phenix^39^ and iterative model building using COOT^40^. Crystallographic refinement statistics are presented in Table S1. All structural figures were prepared using PyMOL (www.pymol.org/).

### Accession codes

PDB ID codes 5OW9, 5OWD, 5OW7, 5MX7 and 2HC4 for co-crystals of 20*S*(OH)D_3_, **4**, **33** 1,20*S*(OH)_2_D_3_ and 1,25(OH)_2_D_3_ in complex with VDR ligand binding domain.

## Electronic supplementary material

Supporting information
