# Supplementary material for: Investigation of 20S-hydroxyvitamin D3 analogs and their 1α-OH derivatives as potent vitamin D receptor agonists with anti-inflammatory activities
Source: Sci Rep. 2018 Jan 24;8:1478. doi: 10.1038/s41598-018-19183-7 (PMC5784132; doi:10.1038/s41598-018-19183-7)
Supplement: Supplementary file 1 — Supporting information [file 41598_2018_19183_MOESM1_ESM.docx]

**Supplementary Information**

**Experimental procedures and characterization of compounds**

**Investigation of 20*S*-hydroxyvitamin D_3_ analogs and their 1α-OH derivatives as potent vitamin D receptor agonists with anti-inflammatory activities**

Zongtao Lin^1,8^, Srinivasa R. Marepally^1^, Emily S. Y. Goh^2^, Chloe Y. S. Cheng^2^, Zorica Janjetovic^3^, Tae-Kang Kim^3^, Duane D. Miller^1^, Arnold E. Postlethwaite^4,5^ , Andrzej T. Slominski^3,6^, Robert C. Tuckey^2^, Carole Peluso-Iltis^7^, Natacha Rochel^7,^*, Wei Li^1,^*

1. Department of Pharmaceutical Sciences, University of Tennessee Health Science Center, Memphis, TN 38163, United States.

2. School of Molecular Sciences, University of Western Australia, Perth, WA 6009, Australia.

3. Department of Dermatology, University of Alabama at Birmingham, Birmingham, AL 35294, United States.

4. Department of Medicine, University of Tennessee Health Science Center, Memphis, TN 38163, United States.

5. Department of Veterans Affairs Medical Center, Memphis TN 38104, United States.

6. VA Medical Center at Birmingham, Birmingham, AL 35294, United States.

7. Department of Integrative Structural Biology, Institute of Genetics and of Molecular and Cellular Biology, Centre National de la Recherche Scientifique, Institut National de la Santé de la Recherche Médicale, Université de Strasbourg, 1 rue Laurent Fries, Illkirch 67404, France.

8. Pressent address : Department of Chemistry, University of Pennsylvania, Philadelphia, PA 19104, United States.

***Corresponding Authors.** Phone: +1 (901) 448 7532. Fax: +1 (901) 448 6828. E-mail: wli@uthsc.edu. Address: 881 Madison Avenue, Room 561, Memphis, TN 38163, United States. (W.L.)

Phone: +33 369 485 293. E-mail: rochel@igbmc.fr. Address: IGBMC, 1 rue Laurent Fries, 67404, Illkirch, France. (N.R.)

Contents

[**1.** **Synthetic procedures.** 2](#_Toc493691868)

[**1.1.** **Synthesis of compounds 4 and 5.** 2](#_Toc493691869)

[**1.2.** **Synthesis of compounds 13 and 14.** 3](#_Toc493691870)

[**1.3.** **Synthesis of compounds 23 and 24.** 5](#_Toc493691871)

[**1.4.** **Synthesis of compound 33.** 7](#_Toc493691872)

[**2.** **Chromatograms for CYP24A1 metabolism of analogs.** 10](#_Toc493691873)

[**3.** **Overlay of co-crystal structures.** 11](#_Toc493691874)

[**4.** **Detailed crystallographic parameters.** 12](#_Toc493691875)

[**5.** **Spectra of compounds.** 13](#_Toc493691876)

[**6.** **References.**](#_Toc493691877) 42

1. **Synthetic procedures.**
   1. **Synthesis of compounds 4 and 5.**

***(3S,10R,13S,14R,17S)-17-acetyl-10,13-dimethyl-2,3,4,9,10,11,12,13,14,15,16,17-dodecahydro-1H-cyclopenta[a]phenanthren-3-yl acetate.* (2).** To a solution of compound **1** (3.58 g, 10.0 mmol) in benzene–hexane (200 mL, 1:1) was added dibromantin (1.72 g, 6.0 mmol, 0.6 equiv.) and 2,2-azobisisobutyronitrile (68 mg, 0.4 mmol, 0.04 equiv.). The mixture was refluxed for 20 min in a preheated oil bath (100 ◦C) and then placed in an ice bath to cool. Insoluble material was removed by filtration and the filtrate was concentrated to yield a yellow-brown solid. To a solution of this yellow-brown solid in tetrahydrofuran (50 mL) was added tetrabutylammonium bromide (0.8 g, 2.5 mmol, 0.25 equiv.) and stirred for 75 min at room temperature. To this reaction mixture was added tetrabutylammonium fluoride (20 mL of 1.0 M solution in tetrahydrofuran, 20 mmol, 2 equiv.) and the resulting solution was stirred for 50 min. Water was added and the mixture was extracted by ethyl acetate. The organic layer was dried and concentrated. The residual was subjected to flash chromatography (hexane:ethyl acetate= 2:1) to give a white solid (1.28 g). Yield: 36%. ^1^H NMR (400 MHz, Chloroform-d) δ 5.57 (dd, *J* = 5.8, 2.5 Hz, 1H), 5.41 (dt, *J* = 5.6, 2.7 Hz, 1H), 4.77 – 4.60 (m, 1H), 2.63 (t, *J* = 9.0 Hz, 1H), 2.50 (ddd, *J* = 14.4, 5.1, 2.4 Hz, 1H), 2.36 (dtt, *J* = 19.4, 9.9, 4.7 Hz, 1H), 2.29 – 1.98 (m, 11H), 1.98 – 1.28 (m, 9H), 0.94 (s, 3H), 0.57 (s, 3H). MS (ESI) m/z 379.3[M + Na]^+^.

***(3S,10R,13S,14R,17S)-17-((S)-2-hydroxy-6-methylhept-5-en-2-yl)-10,13-dimethyl-2,3,4,9,10,11,12,13,14,15,16,17-dodecahydro-1H-cyclopenta[a]phenanthren-3-ol.*** (**3**). A solution of 5-bromo-2-methyl-2-pentene (300 mg, 3.1 mmol, 4 equiv.) in dry THF (10 mL) was added dropwise to Mg (117 mg, 3.9 mmol, 5 equiv.) and 0.5 mg I_2_ in an argon-purged flask and then stirred for 2 h at 45 °C. To the resulting solution cooled down to 0 °C, compound **2** (277 mg, 0.8 mmol, 1 equiv.) in dry THF (5 mL) under argon was added. The solution was allowed to warm to room temperature and was stirred overnight. The reaction mixture was quenched with aq. NH4Cl (sat.), extracted with EtOAc, the organic layer was washed with brine and water, dried by Na2SO4 and concentrated. The crude material was subject to silica gel column chromatography (hexane:ethyl acetate, 7:3) to give a white solid (280 mg). Yield: 87.6 %. ^1^H NMR (400 MHz, Methanol-d4) δ 5.54 (dd, *J* = 5.8, 2.5 Hz, 1H), 5.39 (dt, *J* = 5.5, 2.7 Hz, 1H), 5.17 – 5.05 (m, 1H), 3.60 – 3.40 (m, 1H), 2.41 (ddd, *J* = 14.4, 4.8, 2.4 Hz, 1H), 2.31 – 2.12 (m, 2H), 2.07 – 1.71 (m, 11H), 1.71 – 1.55 (m, 8H), 1.56 – 1.21 (m, 7H), 1.27 (s, 3H), 0.94 (s, 3H), 0.79 (s, 3H). MS (ESI) m/z 421.4 [M + Na]^+^.

***(S,Z)-3-(2-((1S,3aS,7aS,E)-1-((S)-2-hydroxy-6-methylhept-5-en-2-yl)-7a-methyloctahydro-4H-inden-4-ylidene)ethylidene)-4-methylenecyclohexan-1-ol.*** (**4**). A ethyl ether solution of **3** (30 mg, 2 mg/mL) was purged with argon gas for 10 min and subjected to UVB irradiation for 15 min in a quartz tube at 75 °C, using a Rayonet RPR- 100 photochemical reactor (Branford, CT). After removal of solvents, the residue was dissolved in ethanol (10 mL) and heated under reflux for 3 h to allow the conversion from pre-VD3 to VD3. The mixture was analyzed by an Agilent 1100 HPLC system (Santa Clara, CA) to optimize the analytical conditions, and concentrated to minimum volume of ethanol. The separation was carried out using a preparative HPLC system. The reaction mixture (500 μL) was injected by an autosampler onto a 5 μm Phenomenex Luna-PFP column (250 mm × 21.2 mm) (Torrance, CA) with mobile phase following the gradient (0-30 min, 55-85% MeCN) at a flow rate of 15 mL/min. Fractions containing product was monitored by UV absorbance, collected and freeze-dried (3.6 mg, 12%). ^1^H NMR (400 MHz, Chloroform-d) δ 6.23 (d, *J* = 11.2 Hz, 1H), 6.03 (d, *J* = 11.3 Hz, 1H), 5.16 – 5.01 (m, 2H), 4.82 (d, *J* = 2.5 Hz, 1H), 3.96 (s, 1H), 2.82 (dd, *J* = 12.1, 4.2 Hz, 1H), 2.57 (dd, *J* = 13.2, 3.8 Hz, 1H), 2.49 – 2.34 (m, 1H), 2.29 (dd, *J* = 13.1, 7.5 Hz, 1H), 2.25 – 1.83 (m, 3H), 1.83 – 1.60 (m, 12H), 1.56 (m, 10H), 1.48 – 1.31 (m, 2H), 1.29 (s, 3H), 1.22 (s, 1H), 0.72 (s, 3H). MS (ESI) m/z 421.4[M + Na]^+^. HPLC purity = 98.6%. HRMS (ESI+) m/z 381.3157 [M + H – H2O]^+^ (error: 0.0 ppm)

- 1. **Synthesis of compounds 13 and 14.**

***tert-butyl(((3S,8S,10R,13S,14S,17S)-17-((S)-2-(ethoxymethoxy)-4-isopropoxybutan-2-yl)-10,13-dimethyl-2,3,4,7,8,9,10,11,12,13,14,15,16,17-tetradecahydro-1H-cyclopenta[a]phenanthren-3-yl)oxy)dimethylsilane.*** (**7**). To a stirred solution of intermediate **6** (1.1 g, 2.1 mmol) in 20 mL DMF was added NaH (99 mg, 4.1 mmol, 2.0 equiv.). After 30 min stirring at r.t., the reaction mixture was cooled down to 0 °C and then added 2-bromopropane (1.0 g, 8.2 mmol, 4.0 equiv.) dropwisely. The reaction mixture was allowed to warm up to r.t. and vigorously stirred for 2 h, quenched by H2O (40 mL), extracted by hexane. The organic layer was combined, dried over Na2SO4 and concentrated. The residual was subjected to flash chromatography (hexane:ethyl acetate= 19:1) to give a white solid (1.1 g, 1.9 mmol). Yield: 92%. ^1^H NMR (400 MHz, Chloroform-d) δ 5.31 (dt, *J* = 5.5, 1.7 Hz, 1H), 4.85 – 4.63 (m, 2H), 3.81 – 3.34 (m, 5H), 2.26 (ddd, *J* = 13.5, 11.0, 2.6 Hz, 2H), 2.16 (ddd, *J* = 13.3, 5.1, 2.2 Hz, 3H), 2.05 (dt, *J* = 12.3, 3.4 Hz, 2H), 2.01 – 1.91 (m, 1H), 1.92 – 1.76 (m, 3H), 1.76 – 1.38 (m, 8H), 1.33 (s, 3H), 1.25 – 1.11 (m, 10H), 1.11 – 1.00 (m, 1H), 0.99 (s, 4H), 0.89 (s, 10H), 0.81 (s, 3H), 0.05 (s, 6H). MS (ESI) m/z 599.4 [M + Na]^+^.

***(3S,8S,10R,13S,14S,17S)-17-((S)-2-(ethoxymethoxy)-4-isopropoxybutan-2-yl)-10,13-dimethyl-2,3,4,7,8,9,10,11,12,13,14,15,16,17-tetradecahydro-1H-cyclopenta[a]phenanthren-3-ol.*** (**8**). To a solution of ether **7** (810 mg, 1.4 mmol) in THF (10 mL) was added tetrabutylammonium fluoride (1.0 M in THF, 2.8 mL, 2.0 equiv.) and stirred at room temperature for 12 h. The reaction mixture was quenched with sat. NaHCO3 (50 mL), then extracted with ethyl acetate (3 × 20 mL). The combined organic layer was washed with brine (20 mL) and H2O (20 mL), dried over Na2SO4 and dried under reduced pressure. The crude mixture was subjected to flash chromatography (hexane:ethyl acetate=70:30) to give an alcohol with quantitative yield. ^1^H NMR (400 MHz, Chloroform-d) δ 5.35 (dt, *J* = 5.4, 1.8 Hz, 1H), 4.83 – 4.65 (m, 2H), 3.71 – 3.38 (m, 6H), 2.37 – 2.16 (m, 2H), 2.12 – 1.75 (m, 8H), 1.72 – 1.38 (m, 11H), 1.33 (s, 3H), 1.29 – 1.24 (m, 1H), 1.23 – 1.10 (m, 9H), 1.08 (dd, *J* = 12.6, 3.6 Hz, 1H), 1.00 (s, 3H), 0.82 (s, 3H). MS (ESI) m/z 485.4 [M + Na]^+^.

***(3S,8S,10R,13S,14S,17S)-17-((S)-2-(ethoxymethoxy)-4-isopropoxybutan-2-yl)-10,13-dimethyl-2,3,4,7,8,9,10,11,12,13,14,15,16,17-tetradecahydro-1H-cyclopenta[a]phenanthren-3-yl acetate***. (**9**). To a solution of alcohol **8** (560 mg, 1.2 mmol) in DCM (10 mL) was added acetic anhydride (618 mg, 6.1 mmol, 5 equiv.), Et3N (1.2 g, 12.1 mmol, 10 equiv.) and catalytic DMAP (0.05 equiv.). The reaction mixture was stirred at r.t. for 12 h. The solvents were removed under reduced pressure, the resulting mixture was subjected to flash chromatography (10% EtOAc in hexane) to give desired product as a white solid. Yield: 96%. ^1^H NMR (400 MHz, Chloroform-d) δ 5.37 (dd, *J* = 5.0, 1.5 Hz, 1H), 4.89 – 4.45 (m, 3H), 3.79 – 3.27 (m, 5H), 2.46 – 2.24 (m, 2H), 2.10 – 1.78 (m, 10H), 1.71 – 1.37 (m, 11H), 1.33 (s, 3H), 1.30 – 1.05 (m, 11H), 1.01 (s, 3H), 0.99 – 0.84 (m, 1H), 0.82 (s, 3H). MS (ESI) m/z 527.4 [M + Na]^+^.

***(3S,10R,13S,14R,17S)-17-((S)-2-(ethoxymethoxy)-4-isopropoxybutan-2-yl)-10,13-dimethyl-2,3,4,9,10,11,12,13,14,15,16,17-dodecahydro-1H-cyclopenta[a]phenanthren-3-yl acetate***. (**10**). To a solution of compound **9** (400 mg, 0.8 mmol) in benzene–hexane (20 mL, 1:1) was added dibromantin (135 mg, 0.5 mmol, 0.6 equiv.) and 2,2-azobisisobutyronitrile (0.5 mg, 0.03 mmol, 0.04 equiv.). The mixture was refluxed for 20 min in a preheated oil bath (100 ◦C) and then placed in an ice bath to cool. Insoluble material was removed by filtration and the filtrate was concentrated to yield a yellow-brown solid. To a solution of this yellow-brown solid in tetrahydrofuran (20 mL) was added tetrabutylammonium bromide (64 mg, 0.2 mmol, 0.25 equiv.) and stirred for 75 min at room temperature. To this reaction mixture was added tetrabutylammonium fluoride (1.5 mL of 1.0 M solution in tetrahydrofuran, 2 equiv.) and the resulting solution was stirred for 50 min in the dark. Water was added and the mixture was extracted by ethyl acetate. The organic layer was dried and concentrated. The residual was subjected to flash chromatography (hexane:ethyl acetate= 19:1) to give a white solid (151 mg). Yield: 38%. ^1^H NMR (400 MHz, Chloroform-d) δ 5.57 (dd, *J* = 5.7, 2.5 Hz, 1H), 5.40 (dt, *J* = 5.6, 2.7 Hz, 1H), 4.89 – 4.45 (m, 2H), 3.79 – 3.27 (m, 6H), 2.56 – 2.24 (m, 2H), 2.20 – 1.78 (m, 11H), 1.71 – 1.37 (m, 8H), 1.33 (s, 3H), 1.30 – 1.05 (m, 9H), 1.01 (s, 3H), 0.99 – 0.84 (m, 1H), 0.82 (s, 3H). MS (ESI) m/z 525.5 [M + Na]^+^.

***(3S,10R,13S,14R,17S)-17-((S)-2-hydroxy-4-isopropoxybutan-2-yl)-10,13-dimethyl-2,3,4,9,10,11,12,13,14,15,16,17-dodecahydro-1H-cyclopenta[a]phenanthren-3-yl acetate***. (**11**). To 140 mg of compound **10** (0.28 mmol) was added in dry MeOH (5.0 mL) and cooled to at 0 °C. (-)-CSA (64 mg, 0.28 mmol, 1 equiv.) was then added, and the mixture was stirred at room temperature overnight. The reaction was quenched by adding a saturated aqueous solution of NaHCO3 (10 mL). The aqueous layer was extracted with EtOAc. The combined organic fractions were dried over Na2SO4, filtered, and concentrated to give a crude mixture, which was purified by column chromatography using 10% EtOAc in hexane to give product as a white solid (100 mg, 0.23 mmol). Yield: 81%. ^1^H NMR (400 MHz, Chloroform-d) δ 5.57 (dd, *J* = 5.7, 2.5 Hz, 1H), 5.40 (dt, *J* = 5.6, 2.7 Hz, 1H), 4.70 (tt, *J* = 11.5, 4.7 Hz, 1H), 3.72 (ddd, *J* = 10.3, 9.3, 3.8 Hz, 1H), 3.64 – 3.50 (m, 2H), 2.50 (ddd, *J* = 14.4, 5.0, 2.3 Hz, 1H), 2.36 (t, *J* = 12.3 Hz, 1H), 2.21 (ddd, *J* = 12.7, 4.8, 2.5 Hz, 1H), 2.12 – 1.64 (m, 11H), 1.65 – 1.23 (m, 12H), 1.16 (dd, *J* = 7.8, 6.1 Hz, 6H), 0.95 (s, 3H), 0.78 (s, 3H). MS (ESI) m/z 467.4 [M + Na]^+^.

***(3S,10R,13S,14R,17S)-17-((S)-2-hydroxy-4-isopropoxybutan-2-yl)-10,13-dimethyl-2,3,4,9,10,11,12,13,14,15,16,17-dodecahydro-1H-cyclopenta[a]phenanthren-3-ol***. (**12**). To a stirred solution of **11** (100 mg, 0.23 mmol) in methanol (10 mL) was added aqueous solution of KOH (63 mg, 1.1 mmol, 5 equiv.) dissolved in H2O (1 mL). The reaction mixture was stirred for 2 h at room temperature in the dark. Then sat. NH4Cl (10 mL) was added, and methanol was removed by rotary evaporator under reduced pressure. The residue was extracted with ethyl acetate which was then washed with water (10 mL), dried over Na2SO4 and concentrated. The crude mixture was subjected to flash chromatography (hexanes:ethyl acetate = 70:30) to afford product as a white solid (84 mg, 0.2 mmol, 93%). ^1^H NMR (400 MHz, Methanol-d4) δ 5.53 (dd, *J* = 5.7, 2.5 Hz, 1H), 5.37 (dt, *J* = 5.6, 2.7 Hz, 1H), 4.60 (s, 1H), 3.70 – 3.39 (m, 4H), 2.39 (ddd, *J* = 14.3, 4.7, 2.4 Hz, 1H), 2.31 – 2.10 (m, 2H), 2.01 – 1.67 (m, 10H), 1.67 – 1.36 (m, 4H), 1.38 – 1.21 (m, 6H), 1.23 – 1.07 (m, 6H), 0.93 (s, 3H), 0.77 (s, 3H). MS (ESI) m/z 425.4 [M + Na]+. HRMS (ESI+) m/z 385.3108 [M + H – H2O]^+^ (error: 0.3 ppm).

***(S,Z)-3-(2-((1S,3aS,7aS,E)-1-((S)-2-hydroxy-4-isopropoxybutan-2-yl)-7a-methyloctahydro-4H-inden-4-ylidene)ethylidene)-4-methylenecyclohexan-1-ol***. (**13**). An ethyl ether solution of **12** (24 mg, 2 mg/mL) was subjected to UVB irradiation for 15 min in a quartz tube at 75 °C, using a Rayonet RPR- 100 photochemical reactor (Branford, CT). After removal of ethyl ether, the residue was dissolved in ethanol (12 mL) and heated under reflux for 3 h to allow the conversion from pre-VD3 structure to VD3 structure. The mixture was analyzed by an Agilent 1100 HPLC system (Santa Clara, CA) to optimize the analytical conditions, and concentrated to minimum volume of ethanol. The real separation was carried out on a preparative HPLC system. The reaction mixture (500 μL) was injected by an autosampler onto a 5 μm Phenomenex Luna-PFP column (250 mm × 21.2 mm) (Torrance, CA) with mobile phase of acetonitrile-water at a flow rate of 15 mL/min using the following gradient: 0-30 min, 50-70% MeCN. Fractions containing VD3 were collected and freeze-dried (2.9 mg, 12%). ^1^H NMR (400 MHz, Chloroform-d) δ 6.23 (d, *J* = 11.3 Hz, 1H), 6.04 (d, *J* = 11.3 Hz, 1H), 5.10 – 4.99 (m, 1H), 4.82 (d, *J* = 2.5 Hz, 1H), 3.95 (s, 1H), 3.72 (td, *J* = 9.9, 3.8 Hz, 1H), 3.65 – 3.49 (m, 2H), 2.82 (d, *J* = 12.6 Hz, 1H), 2.58 (d, *J* = 13.0 Hz, 1H), 2.39 (dd, *J* = 15.0, 6.8 Hz, 1H), 2.29 (dd, *J* = 13.2, 7.4 Hz, 1H), 2.24 – 1.42 (m, 13H), 1.41 – 1.21 (m, 8H), 1.16 (dd, *J* = 7.4, 6.1 Hz, 6H), 0.71 (s, 3H). MS (ESI) m/z 425.4 [M + Na]^+^. HPLC purity = 96.7%. HRMS (ESI+) m/z 385.3107 [M + H – H2O]+ (error: 0.0 ppm).

- 1. **Synthesis of compounds 23 and 24.**

***(6S)-6-((3S,8S,10R,13S,14S,17S)-3-((tert-butyldimethylsilyl)oxy)-10,13-dimethyl-2,3,4,7,8,9,10,11,12,13,14,15,16,17-tetradecahydro-1H-cyclopenta[a]phenanthren-17-yl)-6-(ethoxymethoxy)-2-methylheptan-3-one***. (**16**). To a solution of alcohol **15** (1.5 g, 2.5 mmol) in anhydrous DCM (50 mL) was added PDC (4.6 g, 12.4 mmol, 5 equiv.) at r.t. The reaction mixture was stirred for 36 h, filtered and concentrated. The residue was added to a hexane wetted silica gel (50 g) column, washed with 20% ethyl acetate in hexanes. The eluent was combined and concentrated to afford ketone (1.4 g, 95%) as a colorless sticky solid. ^1^H NMR (400 MHz, Chloroform-d) δ 5.31 (dt, *J* = 5.6, 1.7 Hz, 1H), 4.78 – 4.64 (m, 2H), 3.70 – 3.38 (m, 3H), 2.61 (p, *J* = 6.9 Hz, 1H), 2.52 – 2.43 (m, 2H), 2.31 – 2.12 (m, 2H), 2.07 – 1.91 (m, 2H), 1.91 – 1.75 (m, 6H), 1.76 – 1.67 (m, 1H), 1.66 – 1.40 (m, 11H), 1.26 (s, 3H), 1.18 (t, *J* = 7.1 Hz, 3H), 1.09 (dd, *J* = 6.9, 0.7 Hz, 6H), 0.99 (s, 3H), 0.88 (s, 9H), 0.79 (s, 3H), 0.05 (s, 6H). MS (ESI) m/z 611.4 [M + Na]^+^.

***(6S)-6-(ethoxymethoxy)-6-((3S,8S,10R,13S,14S,17S)-3-hydroxy-10,13-dimethyl-2,3,4,7,8,9,10,11,12,13,14,15,16,17-tetradecahydro-1H-cyclopenta[a]phenanthren-17-yl)-2-methylheptan-3-one***. (**17**). To a solution of ketone **16** (1.1 g, 1.7 mmol) in THF (10 mL) was added tetrabutylammonium fluoride (1.0 M in THF, 3.4 mL, 2.0 equiv.) and stirred at room temperature for 12 h. The reaction mixture was quenched with sat. NaHCO3 (30 mL), then extracted with ethyl acetate (3 × 20 mL). The combined organic layer was washed with brine (20 mL) and H2O (20 mL), dried over Na2SO4 and dried under reduced pressure. The crude mixture was subjected to flash chromatography (hexane:ethyl acetate=70:30) to give an alcohol with quantitative yield. ^1^H NMR (400 MHz, Chloroform-d) δ 5.35 (dt, *J* = 5.2, 1.8 Hz, 1H), 4.77 – 4.66 (m, 2H), 3.69 – 3.46 (m, 3H), 2.61 (h, *J* = 6.9 Hz, 1H), 2.54 – 2.41 (m, 2H), 2.35 – 2.18 (m, 2H), 2.10 – 1.92 (m, 2H), 1.93 – 1.76 (m, 5H), 1.73 – 1.39 (m, 11H), 1.27 (s, 3H), 1.19 (t, *J* = 7.1 Hz, 4H), 1.10 (dd, *J* = 6.9, 0.7 Hz, 7H), 1.01 (s, 3H), 0.97 – 0.87 (m, 1H), 0.81 (s, 3H). MS (ESI) m/z 497.4 [M + Na]^+^.

***(3S,8S,10R,13S,14S,17S)-17-((S)-2-(ethoxymethoxy)-6-methyl-5-oxoheptan-2-yl)-10,13-dimethyl-2,3,4,7,8,9,10,11,12,13,14,15,16,17-tetradecahydro-1H-cyclopenta[a]phenanthren-3-yl acetate***. (**18**). To a solution of alcohol **17** (645 mg, 1.4 mmol) in DCM (10 mL) was added acetic anhydride (693 mg, 6.8 mmol, 5 equiv.), Et3N (1.4 g, 13.6 mmol, 10 equiv.) and catalytic DMAP (0.05 equiv.). The reaction mixture was stirred at r.t. for 12 h. The solvents were removed under reduced pressure, the resulting mixture was subjected to flash chromatography (10% EtOAc in hexane) to give desired product as a white solid (674 mg, 1.3 mmol). Yield: 96%. ^1^H NMR (400 MHz, Chloroform-d) δ 5.38 (dd, *J* = 4.7, 1.6 Hz, 1H), 4.79 – 4.66 (m, 2H), 4.66 – 4.54 (m, 1H), 3.70 – 3.47 (m, 2H), 2.62 (p, *J* = 6.9 Hz, 1H), 2.48 (dd, *J* = 8.9, 7.0 Hz, 2H), 2.32 (dd, *J* = 8.7, 2.7 Hz, 2H), 2.03 (s, 5H), 2.03 – 1.94 (m, 1H), 1.92 – 1.77 (m, 5H), 1.71 – 1.37 (m, 11H), 1.27 (s, 3H), 1.19 (t, *J* = 7.1 Hz, 3H), 1.10 (dd, *J* = 6.9, 0.7 Hz, 6H), 1.02 (s, 3H), 0.99 – 0.86 (m, 1H), 0.81 (s, 3H). MS (ESI) m/z 539.3 [M + Na]^+^.

***(3S,8S,10R,13S,14S,17S)-17-((S)-2-(ethoxymethoxy)-5,5-difluoro-6-methylheptan-2-yl)-10,13-dimethyl-2,3,4,7,8,9,10,11,12,13,14,15,16,17-tetradecahydro-1H-cyclopenta[a]phenanthren-3-yl acetate***. (**19**). To a solution of ketone **18** (674 mg, 1.3 mmol) in DCM (20 mL) was added (diethylamino)sulfur trifluoride (6.3 mg, 39 mmol, 20 equiv.) at r.t. After being stirred at 80 °C for 3 days, diluted with 50 mL of DCM and then slowly added into ice water. The organic layer was collected, and the aqueous layer was extracted with DCM. The combined organic layer was dried over Na2SO4 and concentrated. The residue was purified by column chromatography on a silica gel (EtOAc:hexanes = 5:95) to give desired product (210 mg, 30%) as light yellowish solid. ^1^H NMR (400 MHz, Chloroform-d) δ 5.45 – 5.32 (m, 1H), 4.81 – 4.66 (m, 2H), 4.60 (tdd, *J* = 11.1, 7.0, 4.2 Hz, 1H), 3.60 (ddq, *J* = 32.8, 9.4, 7.1 Hz, 2H), 2.31 (dd, *J* = 9.0, 2.7 Hz, 2H), 2.12 – 1.91 (m, 6H), 1.93 – 1.70 (m, 7H), 1.71 – 1.38 (m, 9H), 1.30 (s, 3H), 1.28 – 1.05 (m, 6H), 1.05 – 0.98 (m, 8H), 0.98 – 0.82 (m, 2H), 0.81 (s, 3H). MS (ESI) m/z 561.3 [M + Na]^+^.

***(3S,10R,13S,14R,17S)-17-((S)-2-(ethoxymethoxy)-5,5-difluoro-6-methylheptan-2-yl)-10,13-dimethyl-2,3,4,9,10,11,12,13,14,15,16,17-dodecahydro-1H-cyclopenta[a]phenanthren-3-yl acetate***. (**20**). To a solution of compound **19** (220 mg, 0.4 mmol) in benzene–hexane (20 mL, 1:1) was added dibromantin (75 mg, 0.26 mmol, 0.65 equiv.) and 2,2-azobisisobutyronitrile (2.6 mg, 0.02 mmol, 0.04 equiv.). The mixture was refluxed for 20 min in a preheated oil bath (100 ◦C) and then placed in an ice bath to cool. Insoluble material was removed by filtration and the filtrate was concentrated to yield a yellow-brown solid. To a solution of this yellow-brown solid in tetrahydrofuran (20 mL) was added tetrabutylammonium bromide (32 mg, 0.1 mmol, 0.25 equiv.) and stirred for 75 min at room temperature. To this reaction mixture was added tetrabutylammonium fluoride (0.8 mL of 1.0 M solution in tetrahydrofuran, 2 equiv.) and the resulting solution was stirred for 50 min in the dark. Water was added and the mixture was extracted by ethyl acetate. The organic layer was dried and concentrated. The residual was subjected to flash chromatography (hexane:ethyl acetate= 95:5) to give a white solid (110 mg). Yield: 50%. ^1^H NMR (400 MHz, Chloroform-d) δ 5.57 (dd, *J* = 5.7, 2.4 Hz, 1H), 5.40 (dt, *J* = 5.5, 2.7 Hz, 1H), 4.82 – 4.65 (m, 3H), 3.60 (ddq, *J* = 33.5, 9.4, 7.1 Hz, 2H), 2.50 (ddd, *J* = 14.4, 4.9, 2.1 Hz, 1H), 2.43 – 2.29 (m, 1H), 2.16 – 2.07 (m, 2H), 2.05 (s, 3H), 2.02 – 1.84 (m, 5H), 1.84 – 1.50 (m, 7H), 1.50 – 1.34 (m, 3H), 1.31 (s, 3H), 1.26 (dd, *J* = 7.9, 4.5 Hz, 2H), 1.22 (s, 3H), 1.02 (d, *J* = 6.9 Hz, 6H), 0.95 (s, 3H), 0.92 – 0.80 (m, 1H), 0.75 (s, 3H). ^19^F NMR (376 MHz, Chloroform-d) δ -107.02 – -107.43 (m). MS (ESI) m/z 559.4 [M + Na]^+^.

***(3S,10R,13S,14R,17S)-17-((S)-5,5-difluoro-2-hydroxy-6-methylheptan-2-yl)-10,13-dimethyl-2,3,4,9,10,11,12,13,14,15,16,17-dodecahydro-1H-cyclopenta[a]phenanthren-3-yl acetate***. (**21**). To 110 mg of compound **20** (0.21 mmol) was added in dry MeOH (5.0 mL) and cooled to at 0 oC. (-)-CSA (47 mg, 0.21 mmol, 1 equiv.) was then added, and the mixture was stirred at room temperature overnight. The reaction was quenched by adding a saturated aqueous solution of NaHCO3 (10 mL). The aqueous layer was extracted with EtOAc. The combined organic fractions were dried over Na2SO4, filtered, and concentrated to give a crude mixture, which was purified by column chromatography using 10% EtOAc in hexane to give product as a white solid (77 mg). Yield: 79%. ^1^H NMR (400 MHz, Chloroform-d) δ 5.58 (dd, *J* = 5.8, 2.5 Hz, 1H), 5.41 (dt, *J* = 5.5, 2.7 Hz, 1H), 4.71 (tt, *J* = 11.5, 4.9 Hz, 1H), 2.51 (ddd, *J* = 14.5, 5.0, 2.2 Hz, 1H), 2.36 (ddd, *J* = 14.1, 11.8, 2.2 Hz, 1H), 2.17 (ddd, *J* = 12.7, 4.9, 2.6 Hz, 2H), 2.05 (s, 3H), 2.12 – 1.95 (m, 6H), 1.94 – 1.67 (m, 7H), 1.67 – 1.54 (m, 7H), 1.52 – 1.31 (m, 2H), 1.02 (d, *J* = 6.9 Hz, 6H), 0.95 (s, 3H), 0.79 (s, 3H). MS (ESI) m/z 501.4 [M + Na]^+^.

***(3S,10R,13S,14R,17S)-17-((S)-5,5-difluoro-2-hydroxy-6-methylheptan-2-yl)-10,13-dimethyl-2,3,4,9,10,11,12,13,14,15,16,17-dodecahydro-1H-cyclopenta[a]phenanthren-3-ol***. (**22**). To a stirred solution of **21** (30 mg, 0.06 mmol) in methanol (5 mL) was added aqueous solution of KOH (17 mg, 0.3 mmol, 5 equiv.) dissolved in H_2_O (0.5 mL). The reaction mixture was stirred for 2 h at room temperature in the dark. Then sat. NH_4_Cl (10 mL) was added, and methanol was removed by rotary evaporator under reduced pressure. The residue was extracted with ethyl acetate which was then washed with water (10 mL), dried over Na_2_SO_4_ and concentrated. The crude mixture was subjected to flash chromatography (hexanes:ethyl acetate = 70:30) to afford product as a white solid (26 mg, 96%). ^1^H NMR (400 MHz, Chloroform-d) δ 5.58 (dd, *J* = 5.8, 2.5 Hz, 1H), 5.41 (dt, *J* = 5.6, 2.7 Hz, 1H), 3.63 (d, *J* = 11.3 Hz, 1H), 2.48 (ddd, *J* = 14.4, 4.9, 2.3 Hz, 1H), 2.29 (ddq, *J* = 14.0, 11.6, 2.2 Hz, 1H), 2.17 (ddd, *J* = 12.6, 4.8, 2.6 Hz, 2H), 2.13 – 1.38 (m, 18H), 1.38 – 1.22 (m, 2H), 1.29 (s, 3H), 1.02 (d, *J* = 6.9 Hz, 6H), 0.95 (s, 3H), 0.80 (s, 3H). ^19^F NMR (376 MHz, Chloroform-d) δ -103.69 – -110.27 (m). MS (ESI) m/z 459.3 [M + Na]^+^.

***(S,Z)-3-(2-((1S,3aS,7aS,E)-1-((S)-5,5-difluoro-2-hydroxy-6-methylheptan-2-yl)-7a-methyloctahydro-4H-inden-4-ylidene)ethylidene)-4-methylenecyclohexan-1-ol.*** (**23**). An ethyl ether solution of **22** (26 mg, 2 mg/mL) was subjected to UVB irradiation for 15 min in a quartz tube at 75 °C, using a Rayonet RPR- 100 photochemical reactor (Branford, CT). After removal of ethyl ether, the residue was dissolved in ethanol (12 mL) and heated under reflux for 3 h to allow the conversion from pre-VD3 structure to VD3 structure. The mixture was analyzed by an Agilent 1100 HPLC system (Santa Clara, CA) to optimize the analytical conditions, and concentrated to minimum volume of ethanol. The real separation was carried out on a preparative HPLC system. The reaction mixture (500 μL) was injected by an autosampler onto a 5 μm Phenomenex Luna-PFP column (250 mm × 21.2 mm) (Torrance, CA) with mobile phase of acetonitrile-water at a flow rate of 15 mL/min using the following gradient: 0-30 min, 55-75% MeCN. Fractions containing VD3 were collected and freeze-dried (3.6 mg, 14%). ^1^H NMR (400 MHz, Chloroform-d) δ 6.23 (d, *J* = 11.2 Hz, 1H), 6.04 (d, *J* = 11.3 Hz, 1H), 5.06 (dt, *J* = 2.6, 1.3 Hz, 1H), 4.81 (d, *J* = 2.5 Hz, 1H), 3.95 (dt, *J* = 7.5, 3.8 Hz, 1H), 2.83 (dd, *J* = 12.1, 4.3 Hz, 1H), 2.58 (dd, *J* = 13.2, 3.8 Hz, 1H), 2.41 (ddd, *J* = 13.1, 7.9, 4.7 Hz, 1H), 2.29 (dd, *J* = 13.1, 7.5 Hz, 1H), 2.18 (ddd, *J* = 13.6, 9.4, 4.7 Hz, 1H), 2.13 – 1.45 (m, 19H), 1.36 (td, *J* = 12.8, 4.1 Hz, 1H), 1.27 (s, 3H), 1.02 (d, *J* = 6.9 Hz, 6H), 0.73 (s, 3H). ^19^F NMR (376 MHz, Chloroform-d) δ -107.13 (ddt, *J* = 35.0, 20.2, 14.0 Hz). MS (ESI) m/z 459.3 [M + Na]^+^. HPLC purity = 98.7%. HRMS (ESI+) m/z 419.3115 [M + H – H2O]^+^ (error: 1.2 ppm).

- 1. **Synthesis of compound 33.**

***3-((3S,8S,10R,13S,14S,17S)-3-((tert-butyldimethylsilyl)oxy)-10,13-dimethyl-2,3,4,7,8,9,10,11,12,13,14,15,16,17-tetradecahydro-1H-cyclopenta[a]phenanthren-17-yl)-3-(ethoxymethoxy)butanoic acid***. (**26**). To a stirred solution of previously obtained intermediate **25**^1^ (18.2 g, 15.4 mmol) in MeCN : THF (10: 1, 20 mL) was added NaIO4 (2.1 g, 23.1 mmol, 1.5 equiv.) and NaH2PO4 (0.81 g, 6.8 mmol, 0.44 equiv.) in 10 mL of water, and 30% H2O2 (8.7 mL, 77 mmol, 5 equiv.) at 0 °C. The mixture was stirred for 2 h at room temperature, and monitored by TLC using 20% EtOAc in Hexane. Upon completion, solvent was removed under reduced pressure, and the mixture was added brine (20 mL), extracted by DCM (3 × 20 mL), washed with H2O (20 mL), and dried over Na2SO4. The combined organic solution was subjected to flash chromatography under gradient conditions (0-40% EtOAc in hexane) to afford acid (8.4 g, quantitative yield) as a white solid^2^. ^1^H NMR (400 MHz, Chloroform-*d*) δ 10.24 (s, 1H), 5.34 – 5.18 (m, 1H), 4.85 (d, *J* = 7.4 Hz, 1H), 4.68 (d, *J* = 7.4 Hz, 1H), 3.70 – 3.57 (m, 1H), 3.57 – 3.48 (m, 1H), 3.43 (qd, *J* = 10.6, 9.5, 4.2 Hz, 1H), 2.74 – 2.52 (m, 2H), 2.21 (t, *J* = 12.9 Hz, 1H), 2.11 (ddd, *J* = 13.4, 5.1, 2.2 Hz, 1H), 2.01 – 1.85 (m, 2H), 1.85 – 1.70 (m, 2H), 1.70 – 1.62 (m, 2H), 1.62 – 1.53 (m, 1H), 1.53 – 1.45 (m, 2H), 1.44 (s, 1H), 1.43 (s, 3H), 1.39 (d, *J* = 4.2 Hz, 1H), 1.36 (d, *J* = 5.1 Hz, 1H), 1.31 – 1.18 (m, 1H), 1.16 (t, *J* = 7.1 Hz, 3H), 1.13 – 1.02 (m, 1H), 1.02 – 0.95 (m, 1H), 0.94 (s, 3H), 0.91 (d, *J* = 6.6 Hz, 1H), 0.88 – 0.84 (m, 1H), 0.83 (s, 9H), 0.81 – 0.75 (m, 1H), 0.74 (s, 3H), 0.00 (s, 6H). ^13^C NMR (101 MHz, CDCl_3_) δ 173.48, 141.58, 120.97, 89.43, 79.51, 77.21, 72.58, 63.95, 58.14, 56.76, 50.04, 45.80, 42.78, 40.03, 37.36, 36.58, 32.06, 31.75, 31.38, 25.94, 23.77, 23.11, 22.69, 20.86, 19.41, 18.26, 15.06, 13.87, -4.57. HRMS (ESI+) m/z 549.3969 [M + H]^+^ (error: -1.1 ppm).

***3-((3S,8S,10R,13S,14S,17S)-3-((tert-butyldimethylsilyl)oxy)-10,13-dimethyl-2,3,4,7,8,9,10,11,12,13,14,15,16,17-tetradecahydro-1H-cyclopenta[a]phenanthren-17-yl)-3-(ethoxymethoxy)-N-isopropylbutanamide***. (**27**). To a stirred solution of acid **26** (3.4 g, 6.2 mmol) in pyridine (20 mL) was added PyBOP (4.8 g, 9.3 mmol, 1.5 equiv.) and isopropylamine (567 mg, 9.3 mmol, 1.5 equiv.) at 0 °C. The reaction mixture was allowed to warm up to r.t. and stirred for 12 h. Solvent was removed under reduced pressure, and the mixture was added Na2CO3 (1.5 equiv.) in 20 mL H2O and stirred for 0.5 h. EtOAc was used for extraction (3 × 20 mL), the combined solvent was washed with brine (20 mL) and H2O (20 mL), and dried over Na2SO4. The combined organic solution was subjected to flash chromatography under gradient conditions (0-50% EtOAc in hexane) to afford amide (3.4 g, 5.8 mmol, 93%) as a white solid. ^1^H NMR (400 MHz, Chloroform-*d*) δ 6.31 (s, 1H), 5.31 – 5.19 (m, 1H), 4.80 (d, *J* = 6.8 Hz, 1H), 4.64 (d, *J* = 6.9 Hz, 1H), 4.11 – 3.94 (m, 1H), 3.62 (dq, *J* = 9.4, 7.1 Hz, 1H), 3.56 – 3.47 (m, 1H), 3.47 – 3.35 (m, 1H), 2.47 (d, *J* = 14.2 Hz, 1H), 2.41 – 2.35 (m, 1H), 2.28 – 2.14 (m, 1H), 2.11 (ddd, *J* = 13.3, 5.0, 2.1 Hz, 1H), 2.05 – 1.96 (m, 1H), 1.96 – 1.85 (m, 1H), 1.82 – 1.70 (m, 1H), 1.70 – 1.62 (m, 2H), 1.62 – 1.51 (m, 1H), 1.45 (ddq, *J* = 17.9, 7.9, 4.2, 3.5 Hz, 3H), 1.40 – 1.36 (m, 1H), 1.34 (s, 3H), 1.29 (d, *J* = 3.3 Hz, 1H), 1.27 – 1.20 (m, 1H), 1.17 (t, *J* = 7.0 Hz, 3H), 1.10 (s, 3H), 1.08 (s, 3H), 1.06 – 0.96 (m, 2H), 0.94 (s, 3H), 0.92 – 0.88 (m, 3H), 0.83 (s, 9H), 0.82 – 0.77 (m, 1H), 0.73 (s, 3H), 0.00 (s, 6H). ^13^C NMR (101 MHz, CDCl_3_) δ 169.91, 141.61, 121.00, 89.36, 79.98, 77.21, 72.60, 63.56, 58.47, 56.79, 50.11, 47.28, 42.82, 42.75, 41.05, 40.01, 37.36, 36.58, 32.08, 31.77, 31.38, 25.94, 23.81, 23.13, 22.91, 22.70, 22.63, 20.84, 19.41, 18.26, 15.23, 14.00, -4.57. HRMS (ESI+) m/z 588.4820 [M + H]^+^ (error: 1.4 ppm).

***3-(ethoxymethoxy)-3-((3S,8S,10R,13S,14S,17S)-3-hydroxy-10,13-dimethyl-2,3,4,7,8,9,10,11,12,13,14,15,16,17-tetradecahydro-1H-cyclopenta[a]phenanthren-17-yl)-N-isopropylbutanamide***. (**28**). To a solution of amide **27** (3.4 g, 5.7 mmol) in THF (30 mL) was added tetrabutylammonium fluoride (1.0 M in THF, 11.4 mL, 2.0 equiv.) and stirred at room temperature for 12 h. The reaction mixture was quenched with sat. NaHCO3 (50 mL), then extracted with ethyl acetate (3 × 20 mL). The combined organic layer was washed with brine (20 mL) and H2O (20 mL), dried over Na2SO4 and dried under reduced pressure. The crude mixture was subjected to flash chromatography (hexane:ethyl acetate=40:60) to give a secondary alcohol with quantitative yield. ^1^H NMR (400 MHz, Chloroform-*d*) δ 6.36 (s, 1H), 5.29 (dd, *J* = 4.6, 2.6 Hz, 1H), 4.80 (d, *J* = 6.9 Hz, 1H), 4.64 (d, *J* = 6.9 Hz, 1H), 4.09 – 3.96 (m, 1H), 3.62 (dq, *J* = 9.6, 7.1 Hz, 1H), 3.52 – 3.45 (m, 1H), 2.47 (d, *J* = 14.1 Hz, 1H), 2.39 (h, *J* = 7.1, 6.5 Hz, 1H), 2.29 – 2.12 (m, 2H), 2.05 – 1.96 (m, 2H), 1.96 – 1.86 (m, 2H), 1.84 – 1.74 (m, 4H), 1.74 – 1.52 (m, 4H), 1.44 (ddt, *J* = 17.4, 10.2, 3.3 Hz, 3H), 1.37 (d, *J* = 3.6 Hz, 1H), 1.34 (s, 3H), 1.29 (d, *J* = 5.4 Hz, 1H), 1.24 – 1.20 (m, 1H), 1.17 (t, *J* = 7.0 Hz, 3H), 1.12 – 1.10 (s, 3H), 1.08 (s, 3H), 1.06 – 0.96 (m, 1H), 0.95 (s, 3H), 0.90 – 0.81 (m, 1H), 0.74 (s, 3H). ^13^C NMR (101 MHz, CDCl_3_) δ 169.90, 140.81, 121.53, 89.36, 79.97, 77.21, 71.76, 63.56, 58.47, 56.75, 50.02, 47.29, 42.74, 42.29, 41.05, 39.98, 37.24, 36.50, 31.74, 31.66, 31.37, 23.81, 23.12, 22.90, 22.70, 20.86, 19.37, 15.22, 14.00. HRMS (ESI+) m/z 458.3641 [M + H – H2O]^+^ (error: 1.5 ppm).

***(3S,8S,10R,13S,14S,17S)-17-(2-(ethoxymethoxy)-4-(isopropylamino)-4-oxobutan-2-yl)-10,13-dimethyl-2,3,4,7,8,9,10,11,12,13,14,15,16,17-tetradecahydro-1H-cyclopenta[a]phenanthren-3-yl acetate***. (**29**). To a solution of alcohol **28** (1.5 g, 3.2 mmol) in DCM (20 mL) was added acetic anhydride (1.6 g, 15.7 mmol, 5 equiv.), Et3N (1.6 g, 30.1 mmol, 10 equiv.) and catalytic DMAP (0.05 equiv.). The reaction mixture was stirred at r.t. for 12 h. The solvents were removed under reduced pressure, the resulting mixture was subjected to flash chromatography (40% EtOAc in hexane) to give desired product as a white solid (3.0 mmol, 95%). ^1^H NMR (400 MHz, Chloroform-*d*) δ 6.36 (s, 1H), 5.31 (dd, *J* = 4.5, 2.8 Hz, 1H), 4.80 (d, *J* = 6.9 Hz, 1H), 4.64 (d, *J* = 6.9 Hz, 1H), 4.54 (tdd, *J* = 10.7, 6.7, 4.1 Hz, 1H), 4.10 – 3.96 (m, 1H), 3.66 – 3.54 (m, 1H), 3.54 – 3.43 (m, 1H), 2.48 (d, *J* = 14.1 Hz, 1H), 2.38 (d, *J* = 14.1 Hz, 1H), 2.29 – 2.22 (m, 2H), 1.98 (s, 3H), 1.90 (d, *J* = 16.9 Hz, 1H), 1.85 – 1.76 (m, 3H), 1.72 (dd, *J* = 10.6, 6.8 Hz, 1H), 1.69 – 1.63 (m, 2H), 1.63 – 1.52 (m, 1H), 1.52 – 1.46 (m, 1H), 1.46 – 1.40 (m, 2H), 1.39 (d, *J* = 4.4 Hz, 1H), 1.37 – 1.35 (m, 1H), 1.34 (s, 3H), 1.29 (s, 1H), 1.17 (t, *J* = 7.1 Hz, 3H), 1.10 (s, 3H), 1.08 (s, 3H), 1.07 – 1.00 (m, 2H), 0.96 (s, 3H), 0.94 – 0.89 (m, 1H), 0.89 – 0.82 (m, 1H), 0.74 (s, 3H). ^13^C NMR (101 MHz, CDCl_3_) δ 170.50, 169.95, 139.70, 122.46, 89.36, 80.02, 73.93, 63.55, 58.46, 56.67, 49.91, 47.17, 42.71, 41.06, 39.91, 38.10, 36.96, 36.58, 31.72, 31.32, 27.75, 23.79, 23.15, 22.86, 22.70, 22.60, 21.41, 20.78, 19.28, 15.22, 14.02. HRMS (ESI+) m/z 518.3838 [M + H]^+^ (error: -1.3 ppm).

***3-hydroxy-3-((3S,10R,13S,14R,17S)-3-hydroxy-10,13-dimethyl-2,3,4,9,10,11,12,13,14,15,16,17-dodecahydro-1H-cyclopenta[a]phenanthren-17-yl)-N-isopropylbutanamide***. (**32**). To a solution of compound **29** (400 mg, 0.77 mmol) in benzene:hexanes (20 mL, 1:1, v/v) was added dibromantin (133 mg, 0.46 mmol, 0.6 equiv.) and catalytic AIBN (6.1 mg). The mixture was refluxed for 15 min in a preheated oil bath (100 ºC), cooled to 0 ºC, filtered to remove the insoluble material. The filtrate was concentrated to give pale-yellow solid, to which THF (20 mL) and tetrabutylammonium bromide (61.0 mg, 0.19 mmol, 0.25 equiv.) were added. The solution was stirred at r.t. for 75 min, then was added TBAF (0.94 mL, 1.5 M solution in THF, 2 equiv.) and stirred for another 50 min. After addition of water (50 mL), the mixture was extracted with ethyl acetate (3 × 20 mL). The organic layer was combined, dried over Na2SO4 and dried under reduced pressure. The residue was subjected to flash chromatography (hexanes:ethyl acetate) to give impure product. To which in anhydrous MeOH (20 mL) was added CSA (1.0 equiv.) at room temperature and stirred for 12 h in dark. Then K2CO3 (5.0 equiv.) was added to the reaction mixture and stirred for another 12 h. Solvents were removed under reduced pressure, the resulting mixture was added H2O and extracted by EtOAc (3 × 20 mL). The combined organic layer was concentrated and subjected to flash chromatography (hexanes:ethyl acetate=40:60) to give impure product as a white solid. The crude product was analyzed by an Agilent 1100 HPLC system (Santa Clara, CA) to optimize the analytical conditions, and concentrated to minimum volume of ethanol. The real separation was carried out on a preparative HPLC system. The reaction mixture (500 μL) was injected by an autosampler onto a 5 μm Phenomenex Luna-PFP column (250 mm × 21.2 mm) (Torrance, CA) with mobile phase of 45% acetonitrile in water at a flow rate of 15 mL/min. Fractions containing product were monitored by a UV detector at 280 nm, automatically collected, and freeze-dried (70.0 mg, 22%). ^1^H NMR (400 MHz, Methanol-*d*_4_) δ 5.54 (dd, *J* = 5.8, 2.4 Hz, 1H), 5.38 (dt, *J* = 5.5, 2.7 Hz, 1H), 4.59 (s, 1H), 3.97 (p, *J* = 6.6 Hz, 1H), 3.57 – 3.45 (m, 1H), 2.46 – 2.31 (m, 2H), 2.31 – 2.15 (m, 2H), 1.99 – 1.86 (m, 4H), 1.85 – 1.69 (m, 4H), 1.64 (t, *J* = 9.3 Hz, 2H), 1.56 – 1.40 (m, 2H), 1.33 (s, 3H), 1.31 – 1.27 (m, 3H), 1.14 (dd, *J* = 6.6, 1.4 Hz, 6H), 0.94 (s, 3H), 0.79 (s, 3H). HRMS (ESI+) m/z 398.3050 [M + H – H2O]+ (error: -2.2 ppm) and m/z 380.2945 [M + H – H2O]^+^ (error: -4.7 ppm).

***3-hydroxy-3-((1S,3aS,7aS,E)-4-((Z)-2-((S)-5-hydroxy-2-methylenecyclohexylidene)ethylidene)-7a-methyloctahydro-1H-inden-1-yl)-N-isopropylbutanamide***. (**33**). An ethyl ether: EtOH (10:1) solution of **32** (21 mg, 2 mg/mL) was purged with argon gas for 10 min and subjected to UVB irradiation for 15 min in a quartz tube at 75 °C, using a Rayonet RPR- 100 photochemical reactor (Branford, CT). After removal of solvents, the residue was dissolved in ethanol (10 mL) and heated under reflux for 3 h to allow the conversion from pre-VD3 to VD3. The mixture was analyzed by an Agilent 1100 HPLC system (Santa Clara, CA) to optimize the analytical conditions, and concentrated to minimum volume of ethanol. The separation was carried out using a preparative HPLC system. The reaction mixture (500 μL) was injected by an autosampler onto a 5 μm Phenomenex Luna-PFP column (250 mm × 21.2 mm) (Torrance, CA) with mobile phase of 40% acetonitrile in water at a flow rate of 15 mL/min. Fractions containing product was monitored by UV absorbance, collected and freeze-dried (2.1 mg, 10%). ^1^H NMR (400 MHz, Methanol-*d*_4_) δ 6.12 (d, *J* = 11.2 Hz, 1H), 5.93 (d, *J* = 11.2 Hz, 1H), 4.98 – 4.91 (m, 1H), 4.65 (dd, *J* = 2.8, 1.3 Hz, 1H), 4.49 (s, 1H), 3.87 (hept, *J* = 6.6 Hz, 1H), 3.67 (tt, *J* = 8.8, 3.9 Hz, 1H), 2.75 (dd, *J* = 12.1, 4.0 Hz, 1H), 2.49 – 2.38 (m, 1H), 2.36 – 2.29 (m, 1H), 2.26 (d, *J* = 14.2 Hz, 1H), 2.15 (s, 1H), 2.12 (d, *J* = 2.1 Hz, 1H), 2.10 – 2.08 (m, 1H), 2.04 – 1.97 (m, 2H), 1.94 – 1.82 (m, 2H), 1.80 – 1.64 (m, 1H), 1.64 – 1.57 (m, 2H), 1.55 (s, 1H), 1.53 – 1.34 (m, 2H), 1.32 – 1.23 (m, 1H), 1.21 (s, 3H), 1.19 (s, 1H), 1.04 (dd, *J* = 6.6, 1.5 Hz, 6H), 0.85 – 0.72 (m, 1H), 0.59 (s, 3H). ^13^C NMR (101 MHz, MeOD) δ 173.75, 147.02, 142.07, 137.51, 122.55, 119.48, 112.65, 75.20, 70.55, 61.26, 57.76, 48.17, 47.09, 47.01, 42.29, 42.18, 36.60, 33.58, 29.82, 26.88, 24.38, 23.31, 22.98, 22.66, 22.50, 14.04. HPLC purity = 99.1%. HRMS (ESI+) m/z 398.3074 [M + H – H2O]+ (error: 3.7 ppm) and m/z 380.2968 [M + H – H2O]^+^ (error: 3.4 ppm).

1. **Chromatograms for CYP24A1 metabolism of analogs.**





**Supplementary Fig. S1.** Metabolism of 20S(OH)D3 analogs in phospholipid vesicles by rat CYP24A1. Analogues were incorporated into phospholipid vesicles at a ratio of 0.018 mol/mol phospholipid and incubated with rat CYP24A1 (0.14 µM) for 10 min at 37 ˚C. Samples were analyzed by reverse-phase HPLC using an acetonitrile in water gradient as described in the Methods. (A) Chromatogram for control incubation of 20S(OH)D3 where human adrenodoxin was omitted from the reaction. (B) Test reaction for 20S(OH)D3; (C) test reaction for **13**; (D) test reaction for **4**; (E) test reaction for **23**; (F) test reaction for **33**. Arrows indicate major products not present in control chromatograms where adrenodoxin was excluded. RT, retention time in min.

1. **Overlay of co-crystal structures.**


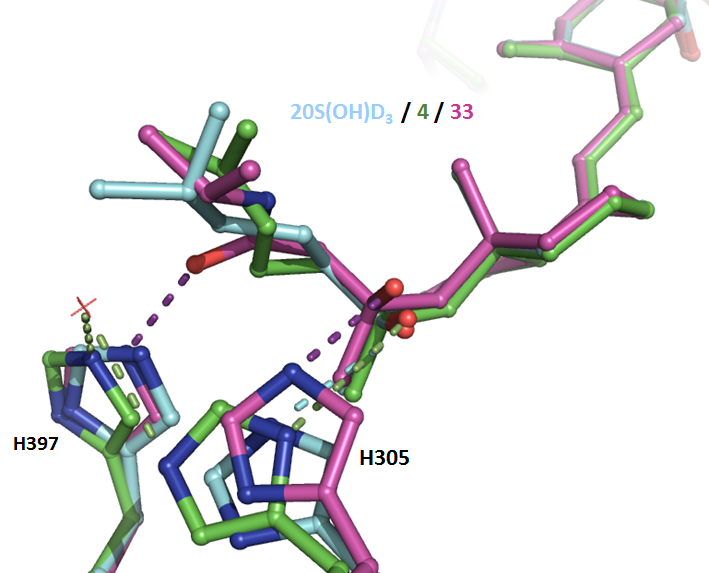


**Supplementary Fig. S2:** Structural comparisons of the VDR LBD around His305 and His397. VDR LBDs in complex with 20S(OH)D_3_, **4** and **33** are superimposed and shown in cyan, green, and pink, respectively.

1. **Detailed crystallographic parameters.**

**Supplementary Table S1: Crystallographic data collection and refinement statistics for zVDR LBD complexes.**

| *VDR/*  *PDB ID* | 20S(OH)D_3_  5OW9 | 4  5OWD | 33  5OW7 |
| --- | --- | --- | --- |
| *Data processing* |  |  |  |
| *Beamline* | ID30 | ID30 | ID30 |
| *X-ray source detector* | PILATUS 6M | PILATUS 6M | PILATUS 6M |
| *λ (Å)* | 0.976251 | 0.976251 | 0.976251 |
| *Resolution (Å)* | 22.74-2.403 | 23.88-2.151 | 21.8-2.1 |
| *Crystal space group* | P 6_5_22 | P 6_5_22 | P 6_5_22 |
| *Cell parameters (Å)* | *a* = *b* = 65.98;  *c* = 262.87 | *a* = *b* = 65.75;  *c* = 262.97 | *a* = *b* = 65.699;  *c* = 262.259 |
| *Unique reflections* | 13900 | 23370 | 20462 |
| *Mean redundancy* | 1.2 | 1.2 | 1.2 |
| *Rsym (%)* | 10.7 | 13.6 | 9.6 |
| *Mean I/σ(I)* | 10.29 | 13.82 | 11.75 |
| *completeness (%)* | 98 | 99 | 99 |
| *Refinement* |  |  |  |
| *rmsd bond length (Å)* | 0.014 | 0.014 | 0.014 |
| *rmsd bond angles (deg)* | 1.67 | 1.53 | 1.62 |
| *Rcryst (%)* | 18.95 | 20.76 | 18.78 |
| *Rfree (%)* | 24.56 | 22.57 | 22.53 |
|  |  |  |  |
| *no. of non-H atoms* |  |  |  |
| *protein* | 2001 | 2006 | 2038 |
| *ligands* | 29 | 29 | 30 |
| *water* | 87 | 81 | 151 |
| *average B factor* |  |  |  |
| *protein* | 49.81 | 56.12 | 41.15 |
| *ligands* | 45.6 | 43.35 | 30.95 |
| *water* | 55.64 | 63.53 | 56.01 |

1. **Spectra of compounds.**

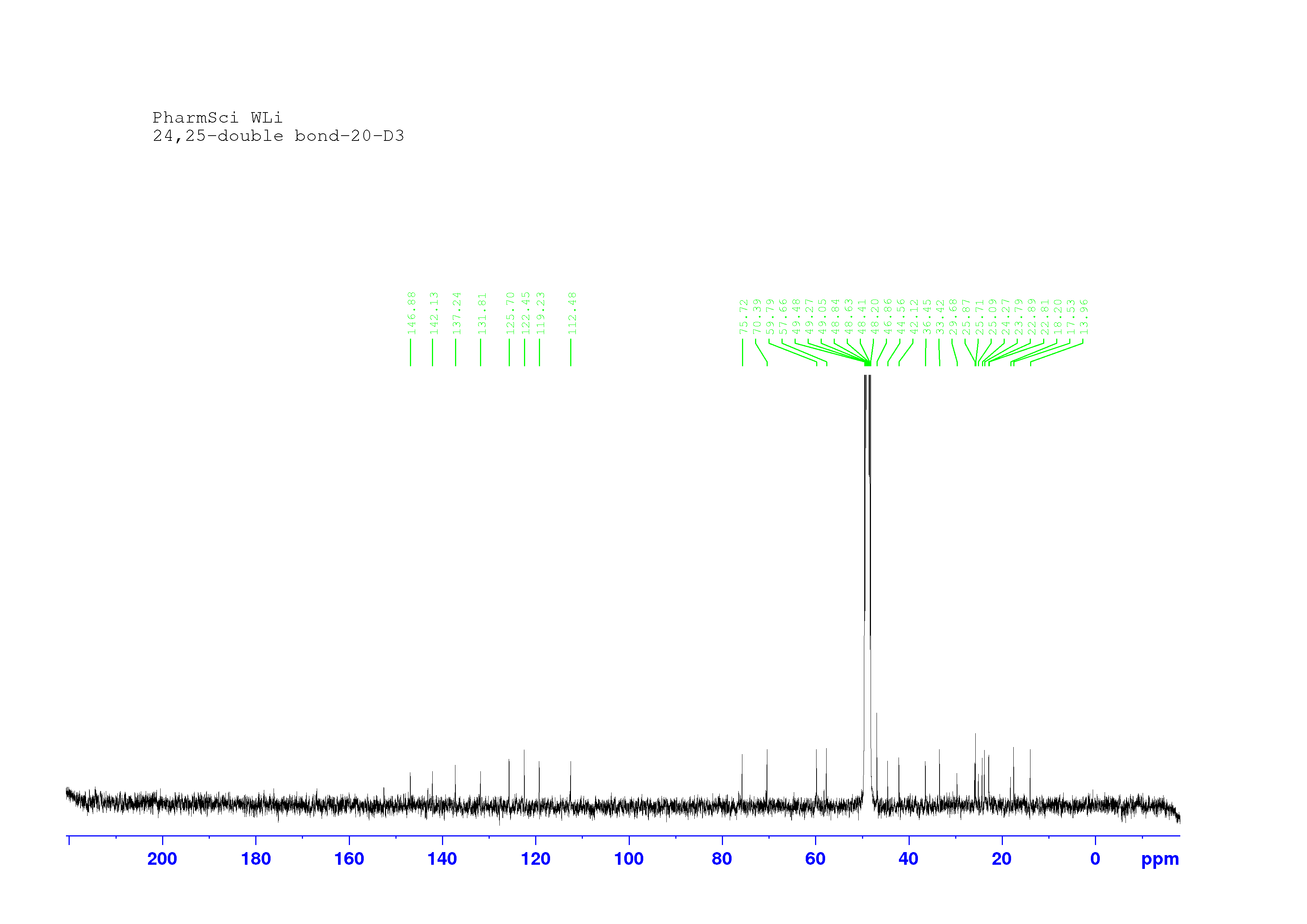


**^13^C-NMR of compound 4.**


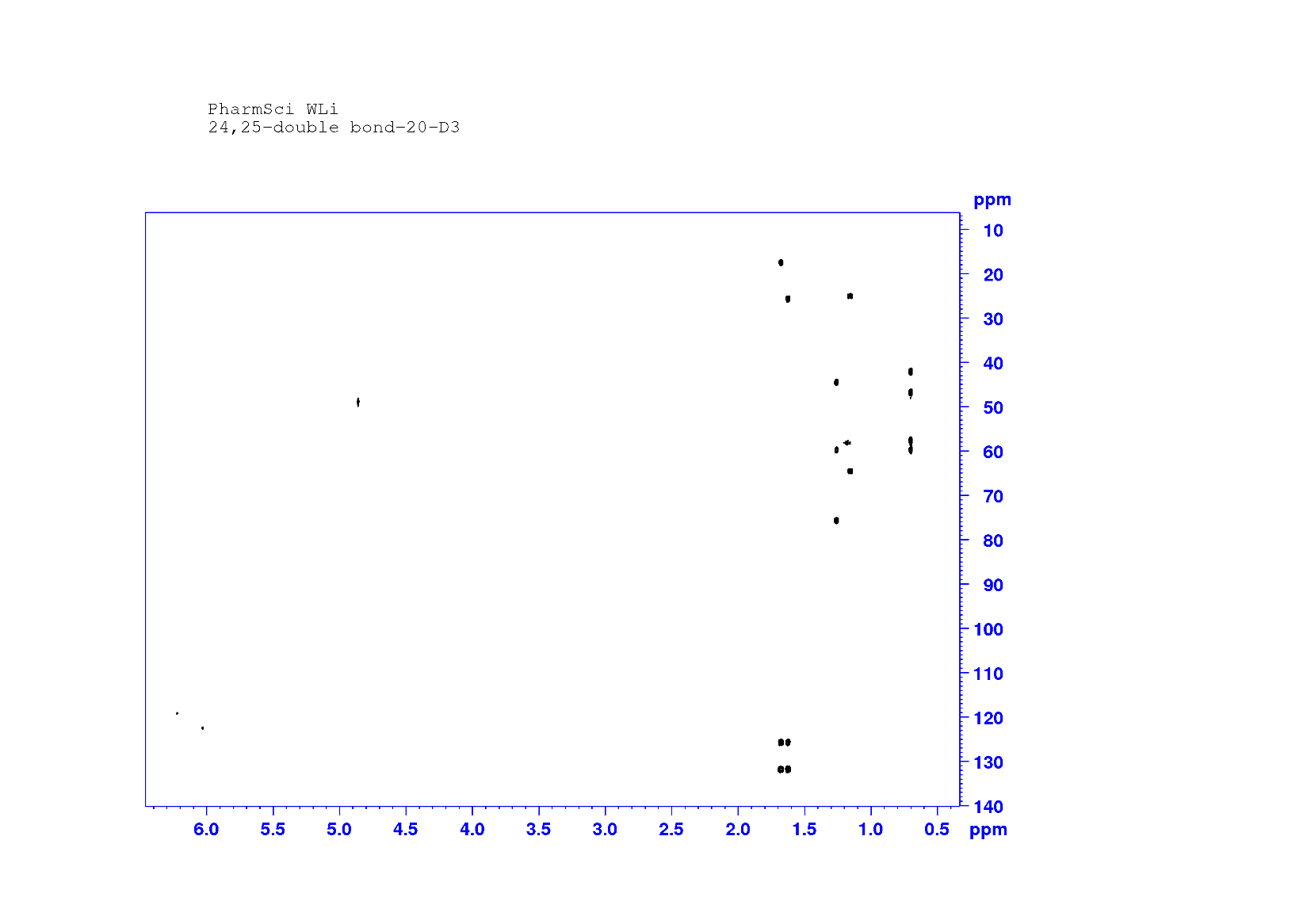


**HMBC of compound 4.**

**
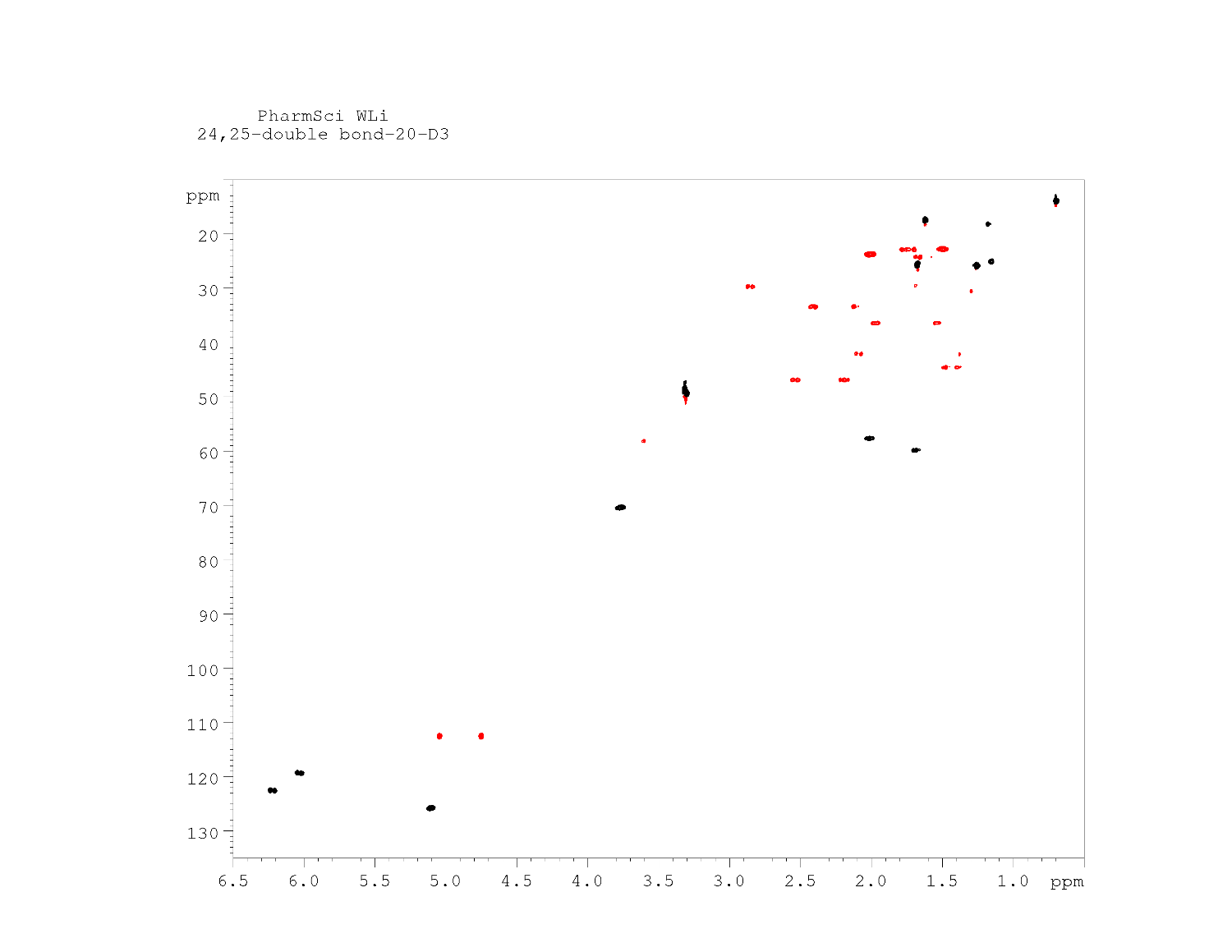
**

**HSQC of compound 4.**

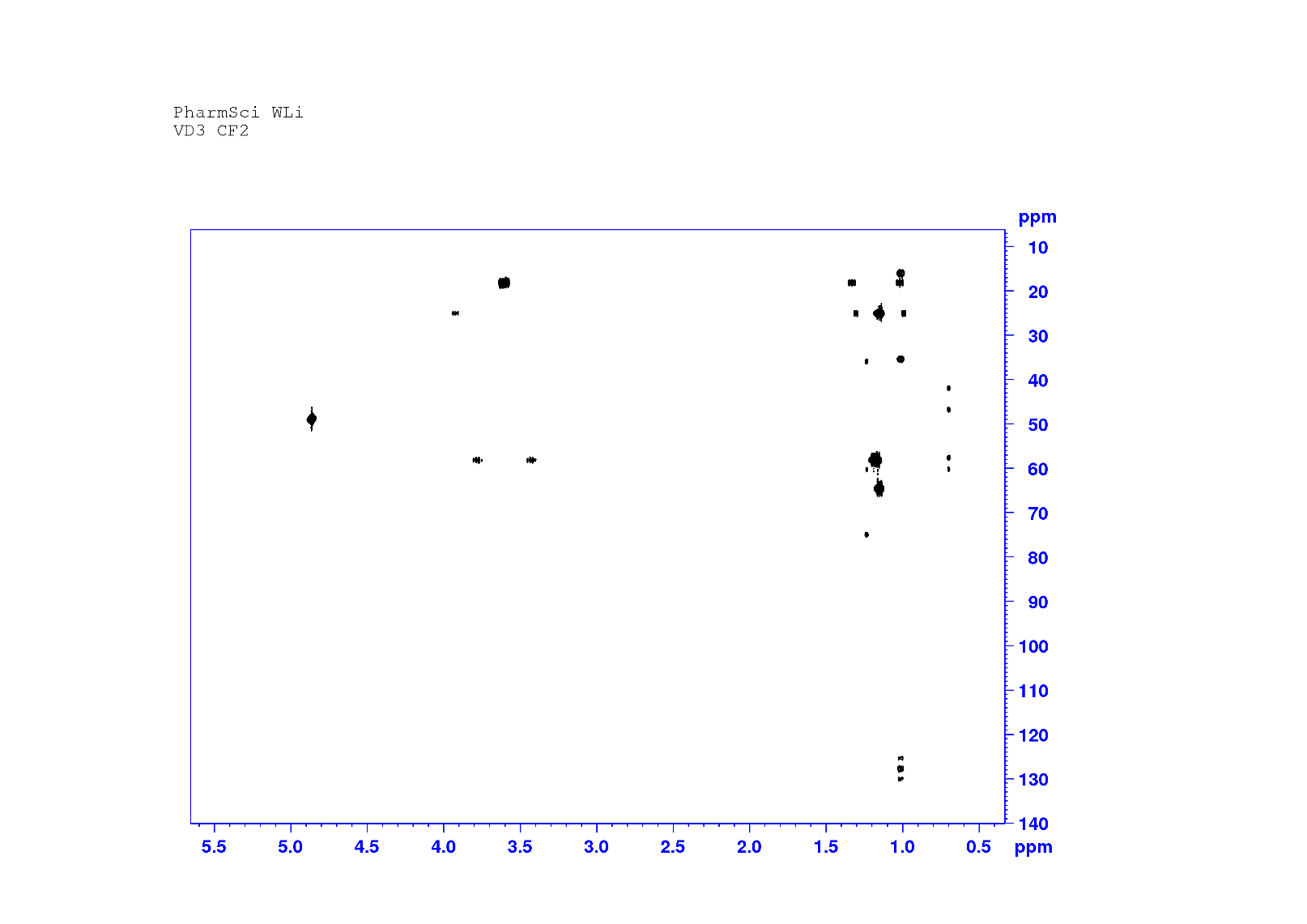


**HMBC of compound 23.**

**
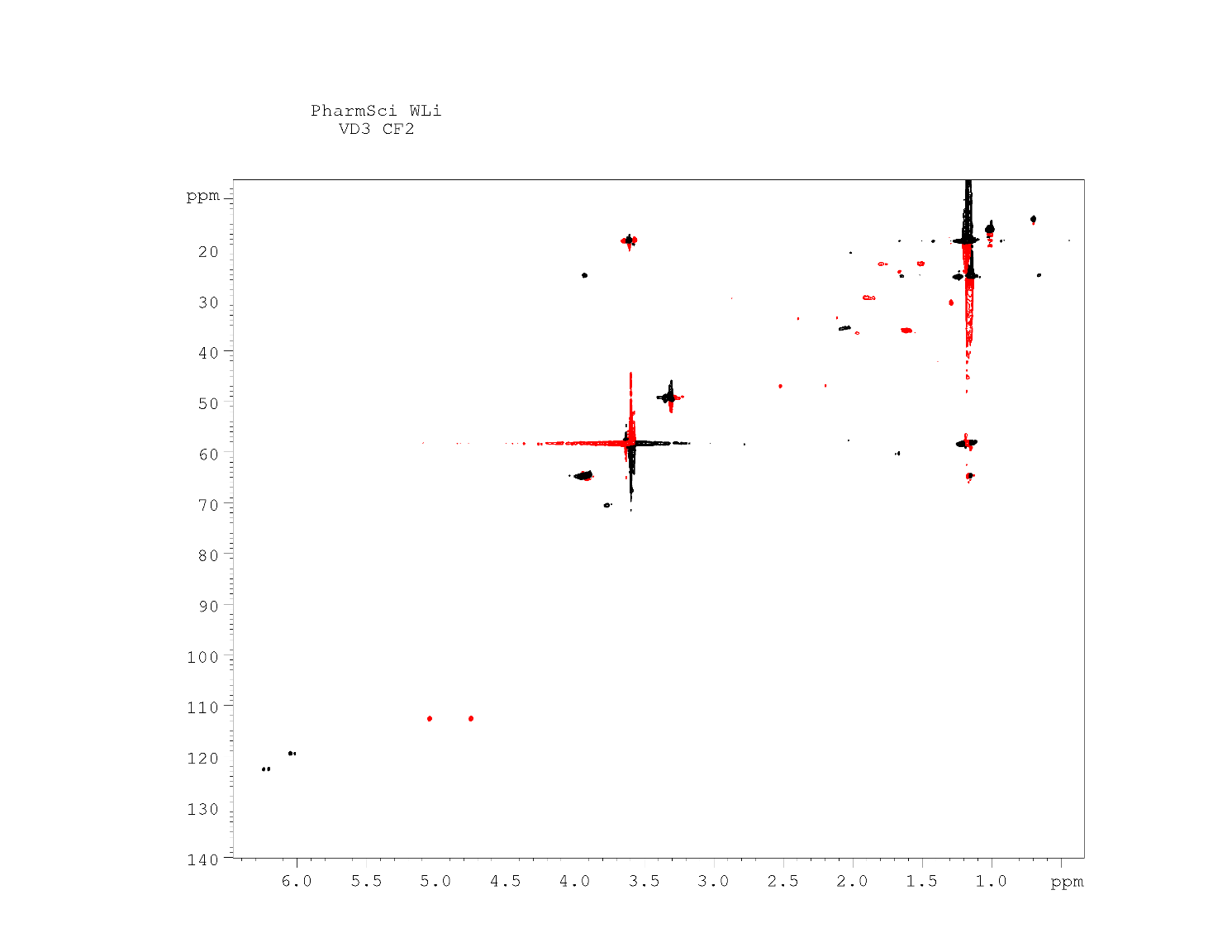
**

**HSQC of compound 23.**

1. **References.**

1. Lin, Z.; Marepally, S. R.; Ma, D.; Kim, T. K.; Oak, A. S.; Myers, L. K.; Tuckey, R. C.; Slominski, A. T.; Miller, D. D.; Li, W. Synthesis and Biological Evaluation of Vitamin D3 Metabolite 20S,23S-Dihydroxyvitamin D3 and Its 23R Epimer. *J Med Chem* 2016, *59*, 5102-5108.

2. Chandrasekhar, S.; Rao, C. L.; Reddy, M. S.; Sharma, G. D.; Kiran, M. U.; Naresh, P.; Chaitanya, G. K.; Bhanuprakash, K.; Jagadeesh, B. Beta-sugar aminoxy peptides as rigid secondary structural scaffolds. *J Org Chem* 2008, *73*, 9443-9446.
